# Widespread gene fusion artifacts in helminth genome annotations

**DOI:** 10.1186/s12864-026-12589-y

**Published:** 2026-02-04

**Authors:** Emma L. Collington, Andrew C. Doxey, Brendan J. McConkey, D. Moira Glerum

**Affiliations:** 1 Department of Biology, University of Waterloo, 200 University Ave W., Waterloo, ON N2L 3G1 Canada; 2 Waterloo Institute for Nanotechnology, University of Waterloo, Waterloo, ON Canada

**Keywords:** Helminth, Nematodes, Platyhelminths, Parasite genomics, Fusion genes, Annotation errors

## Abstract

**Background:**

Current helminth genomes possess thousands of predicted fusion genes, encoding novel protein domain architectures that are unique to these species. To investigate this, we analyzed 20,313 two-domain proteins annotated in current helminth genomes, of which 10,297 are apparently unique to helminths, and used RNA-seq data from 20 species of helminth to examine their plausibility as true fusion genes. For comparison, we analyzed a set of 400 high confidence, evolutionarily conserved domain fusions that are present in both helminth and non-helminth species.

**Results:**

Our analysis suggests that, in contrast to genuine fusion genes, the majority of helminth-specific fusion genes in the 20 species investigated are likely gene prediction artifacts based on several criteria: (1) they show a lack of correlation between RNA-seq derived expression levels of the first and second “fused” domains, as well as the interdomain region; (2) they have significantly longer interdomain regions; (3) there is significantly less continuity of coverage in their interdomain regions consistent with breakpoints in RNA-seq coverage; and (4) they are generally not supported in *de novo* transcriptome assemblies.

**Conclusions:**

Proteins containing novel domain combinations have been included in widely used sequence and protein databases, including WormBase ParaSite and InterPro, but the analyses presented here suggest that many helminth-specific domain fusion proteins are erroneously annotated. These findings emphasize the importance of using RNA-seq data to validate gene predictions in helminth genomes, especially those with unique structures not observed in other species. Given the increasing need to accurately identify helminth-specific proteins as therapeutic targets, the accuracy of proteome annotation in widely used genomic databases is essential.

**Supplementary Information:**

The online version contains supplementary material available at 10.1186/s12864-026-12589-y.

## Background

Infections by parasitic helminths are currently estimated to affect one quarter of the human population and have detrimental impacts on crop and livestock industries [1] with long term socioeconomic impacts [2]. While mass drug administration strategies have been implemented in affected regions, existing anthelminthic agents have side-effects and can be toxic to the host, as well as environmentally damaging. Moreover, an increase in helminth resistance to these agents has made the discovery of new therapeutic targets essential for efforts to control human, livestock, and crop infections [2–4]. Indeed, parasitic infections are increasingly being referred to as ‘re-emerging’ diseases due to new outbreaks in regions where infection levels were previously under control, as a result of the changing environmental and sociopolitical climate [5–7]. For several of the possible approaches to identifying new therapeutic targets, accurate genome and proteome annotations for an increasing variety of helminths is essential.

The genome of the free-living helminth, *Caenorhabditis elegans*, was the first multicellular organism to be sequenced and subsequent efforts have sequenced the genomes of a wide variety of parasitic helminths of interest to human health and agriculture, with many draft genomes now available in online sequence databases [1, 8, 9]. Once sequenced and assembled, helminth genomes undergo extensive automated and manual annotation processes, where gene features within a DNA sequence, such as coding regions, are identified and mapped [10], typically using reference genomes from related species. The *C. elegans* genome has been used to annotate the genomes of other nematodes, although its usefulness is limited due to its free-living life cycle and the amount of species-specific genetic information found in helminths, associated with parasitic life cycles and host environments [1, 8, 11, 12]. Draft genomes can then be used to predict helminth proteomes, which are functionally annotated based on the domains or domain superfamilies found within proteins, using tools such as InterProScan and associated Gene Ontology terms [8, 13–16].

**Fig. 1:**
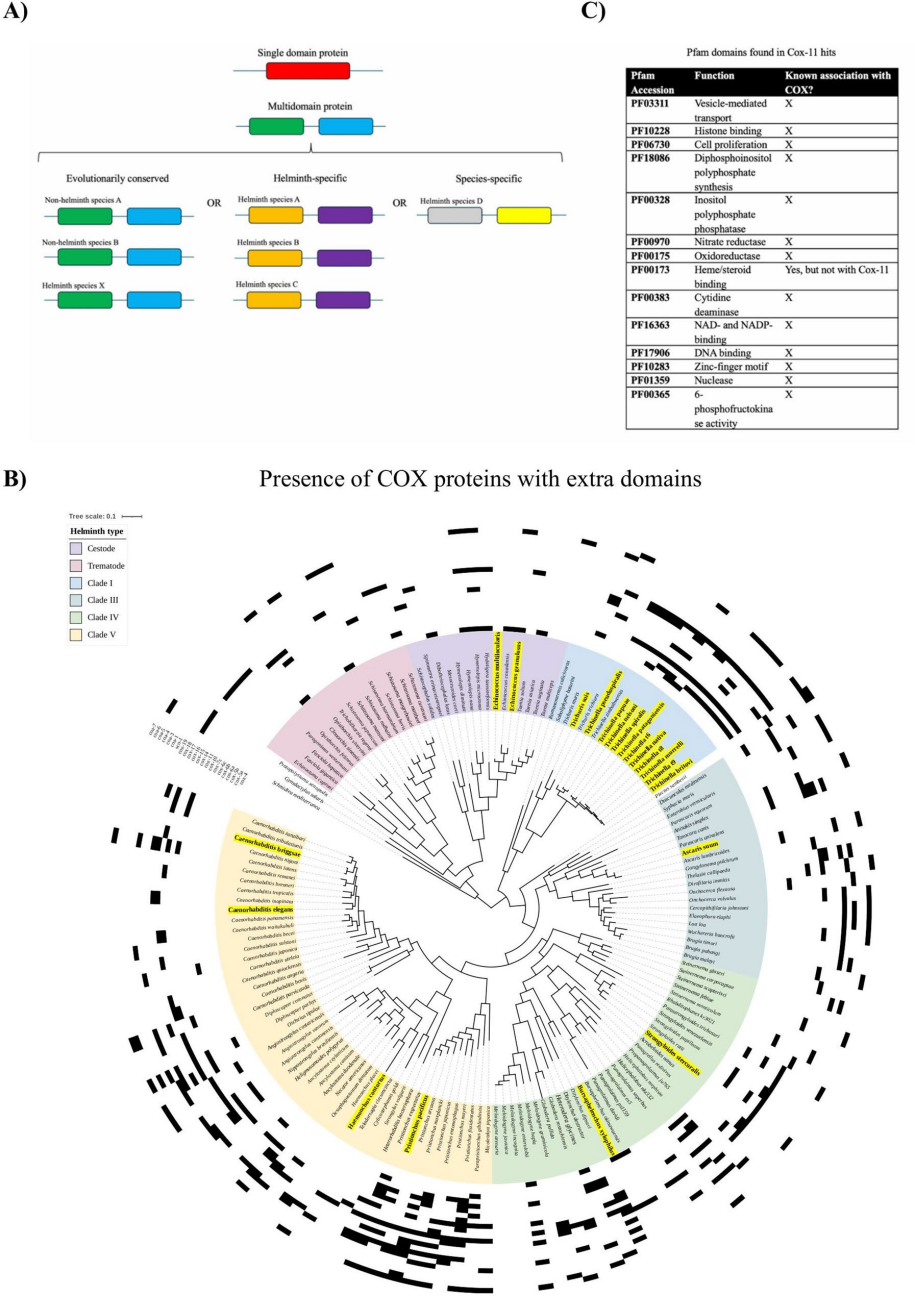
Schematic of domain structures in helminth proteins discussed in this paper. **A** In this work, we analyze (i) Proteins with two domains that are evolutionarily conserved and occur widely in both helminth and non-helminthic species, (ii) Two-domain proteins that are unique to helminths, and (iii) Two-domain proteins that are specific to a single species of helminth. **B** Presence/absence profile showing potential multi-domain ‘hits’ for COX-related proteins, which could represent novel gene fusion events. BLASTP searches identified possible hits for COX subunits and assembly factors: cases where ‘hits’ were identified that did not match the query architecture are shown in black. Proteins of interest include (from inside to outside): cytochrome *c* oxidase subunits Cox-4, Cox-5a, Cox5b, Cox-6a, Cox-6b, Cox-6c, and Cox-7c; COX assembly factors Cox-10, Cox-11, Cox-14, Cox-15, Cox-16, Cox-17, Cox-18, Cox-19, Sco1, Coa-1, Coa-3, Coa-4, Coa-5, Coa-6, and Coa-7. Species for which RNA-seq data were selected are shown highlighted and in bold on the phylogenetic tree. For tree construction methods, please refer to our previous work [26] **C** Sample of additional domains and their associated functions, found in architectures with the COX11 domain. *C. elegans* contains only a single CtaG_Cox11 domain, whereas we identified helminth protein architectures not found outside of helminth taxa that contain additional domains with functions not associated with COX assembly

Protein domains represent fundamental units of protein structure, function, and evolution [17–19] and can exist on their own in single domain proteins, or can occur in larger, more complex multidomain proteins (see Fig. 1A). The amino- to carboxy-terminal order of domains is referred to as the ‘architecture’ of a protein [20–22] and most proteins are modular and have multiple domains, which allows for the generation of complex proteins from a limited overall number of domain structures [23, 24]. Within a multidomain architecture, the function of a given domain is believed to be largely conserved but may be modified or specialized when it occurs in a larger architectural context. Determining the overall function of a multidomain protein requires considering the functions of all the component domains and how they might influence each other [25].

Recent genomic analysis of helminths has identified a surprising number (*n* = 14,596 in 81 species, Coghlan et al. 2019) of apparent fusion genes encoding novel protein domain architectures not observed in other taxa. Possible mechanisms for the acquisition of novel protein domain architectures in helminths include gene fusions, modification of splicing patterns, extension of exons into non-coding regions, exon shuffling, retrotransposition, and horizontal gene transfer [27, 28]. From an evolutionary standpoint, shuffling domain architectures allows new functions to be added to existing proteins without the need for the *de novo* generation of entirely new proteins [29, 30]. For example, the addition of domains to a protein may result in “moonlighting” functions, where a protein can function in multiple biological pathways, and change functional roles depending on subcellular localization, cell type, oligomeric state, and/or concentration of ligands/substrates/cofactors.

Despite the widespread evolutionary role of domain shuffling and gene fusion in creating novel protein domain architectures, there are reasons to be cautious. The accuracy of these predicted fusions is highly dependent on the quality of genome assemblies and annotations being analyzed [31], and unfortunately, there is no universally agreed upon ‘gold standard’ method for these processes in helminths [2, 11, 32]. There are also a number of technical challenges in genome sequencing and assembly that are uniquely associated with helminths: (1) adult life cycle stages occur within hosts, making it difficult to obtain intact and uncontaminated samples, and earlier free-living life cycle stages are microscopic, environmentally resistant, and extraction of sufficient quantities of DNA for sequencing reactions is difficult [10, 32]; and (2) obtaining enough DNA for a sequencing reaction often requires either a whole genome amplification step (which risks the introduction of sequencing bias), or the pooling of information from multiple members of a species, which leads to difficulties in assembly due to the diversity and heterozygosity of helminth populations [32]. Indeed, genome annotation errors have been shown to occur within helminth datasets, including fragmented gene models and genes that have been incorrectly merged [10]. Functional annotations are also subject to error, as sequence divergence can result in a failure to detect conserved domains [16]. Furthermore, an estimated 47% of helminth gene families are currently lacking functional annotations, with many helminth proteins considered ‘hypothetical’ or ‘putative’, and few experimental protocols that can validate the predictions [1, 10, 11, 32].

Given the numerous potential sources of genome annotation error in helminth proteomes and inconsistent biological functions seen in examples of apparent fusion genes (described further in Results), we wondered about the validity of the thousands of domain architectures that are proposed to be unique to helminth proteomes. Building on our previous comparative proteome analysis of helminths [26], we performed a comprehensive analysis of 20,313 two-domain proteins annotated in current helminth genomes, of which 10,297 are helminth-specific. We elected to investigate two-domain proteins as they represent the simplest class of multidomain proteins, which allowed for the investigation of domain organization without the confounding complexity of larger architectures. We measured the extent that these gene models are supported by transcriptomic datasets, comparing helminth-specific fusions to a set of genuine fusion genes that are evolutionarily conserved beyond helminths. Our results strongly suggest that the majority of helminth-specific fusion genes are artifacts and have likely resulted from gene prediction errors, which need to be addressed in future genome annotations for hundreds of helminth species.

## Methods

### RNA-seq data

All RNA-seq data for this project was obtained from the NCBI Sequence Read Archive [33], using the SRA toolkit v.3.0.7 [34] to retrieve 21 previously published helminth RNA-seq datasets [35–43]. Please refer to Supplemental Table S1 for a list of datasets and associated publications. Technical reads and reads that failed to pass quality filtering were removed, and files were converted to FASTQ format. FastQC v.0.12.1 [44] was used for quality control, and only datasets that passed all basic statistics were utilized. Adapter trimming was performed using Trimmomatic v.0.39 [45] and HISAT2 v.2.2.1 [46] was used to align reads to reference coding sequences obtained from WormBase Parasite [8, 9, 47–55]. Samtools v.1.18 [56] was used to produce sorted, indexed .bam files for visualization using IGV v.2.17.1 [57].

### Identification of two-domain proteins unique to helminths

In our previous work [26], PfamScan [58] v1.6 (database 35.0) was used to annotate the proteomes of 155 species of helminth. A list of all architectures in these species was generated and filtered to only include protein architectures with two domains (see Supplemental Table S2, which were individually investigated using InterPro v104.0 [13] to generate a list of two-domain architectures unique to helminths (See Supplemental Table S3). This list was further subdivided into architectures seen in multiple species of helminths (referred to as the helminth-specific dataset, see Supplemental Table S4, and architectures only seen in one species of helminth (referred to as the species-specific dataset, Supplemental Table S5). Transcripts matching these two-domain architectures were then identified in the species with RNA-seq data using our PfamScan annotations.

### Identification of evolutionarily conserved two-domain architectures

We additionally generated a comparison dataset of evolutionarily conserved architectures that contain two-domains. Candidate metabolic proteins were identified from the following KEGG pathways [59]: carbohydrate metabolism, the tricarboxylic acid cycle, the pentose phosphate pathway, the glucuronate pathway, pyruvate metabolism, fatty acid metabolism, energy metabolism, glycerolipid metabolism, steroid metabolism, unsaturated fatty acid metabolism, alpha-linolenic acid metabolism, arachidonic acid metabolism, sphingolipid metabolism, ether lipid metabolism, glycerophospholipid metabolism, pyrimidine metabolism, purine metabolism, and amino acid metabolism. These proteins were then found in *C. elegans* using WormBase [9], and run through PfamScan [58] to identify those with two-domains. To ensure that only evolutionarily conserved proteins were included, these two-domain architectures were checked against InterPro [13], and only architectures found in more than 1000 proteins were retained (see Supplemental Table S6. Transcripts that match these two-domain architectures were then identified from our PfamScan annotations for the 21 species for which we had RNA-seq data (see Supplemental Table S7).

### Comparing evolutionarily conserved two-domain proteins and those unique to helminths

To compare the datasets of two-domain proteins, we extracted the following information from the RNA-seq .bam files for each transcript: transcript length, total number of reads mapping to that transcript, and average read length. Transcripts with fewer than 10 reads overall were dropped from the dataset. PfamScan [58] annotations were used to identify the start and stop positions of the two domains and the interdomain region, and pysam software (version 0.23.3, 60) was used to extract the average read count of these regions, normalized to the length of the region. Read counts were transformed by log(1 + x), and transcripts with 0 reads each in domain one, the interdomain region, and domain two were removed. See Supplementary Table S7 for the dataset of evolutionarily conserved two-domain proteins, Supplementary Table S4 for the helminth-only two-domain proteins, and Supplementary Table S5 for the species-specific proteins.

To assess the correlation between the average read counts in regions of interest, scatterplots were produced for each dataset, comparing average read counts in domain one and domain two. A correlational coefficient and associated *p* value was calculated for each dataset, and Fisher’s *Z*-test was used to compare the correlational coefficients.

The average interdomain lengths from each dataset were additionally compared. Group differences for these three datasets were assessed using a Kruskal-Wallis test, and pairwise comparisons were performed using Tukey’s HSD test with a family-wise error rate correction for multiple comparisons.

RNA-seq profiles were also investigated for places within the architectures of interest where the read coverage drops to zero. This was addressed in two different ways. First, for each position (x) within the interdomain region, the number of reads spanning (x-1) to (x + 1) was counted, and the maximum and minimum values from these were plotted. Correlational coefficients were compared using Fisher’s Z test.

Second, we looked at consecutive base positions for continuity of coverage. For each position between the first and last ten positions in the proteins of interest, the read depth was calculated at that position (x) and the next base (x + 1). The reads spanning these junctions were then divided by the read depth at these positions to produce a ratio: values close to 1 represent continuous coverage at consecutive positions, and a ratio of 0 representing an abrupt change in the number of reads between two consecutive bases. Only proteins with more than 20 reads overall were included to ensure sufficient coverage to identify genuine breakpoints as opposed to gaps in coverage in genes with low expression overall. For the purposes of this investigation, positions with more than five reads at either position, and with a ratio below 0.1, are considered breakpoints. Adjacent breakpoints were collapsed into ranges with their ratios averaged. As the number of breakpoints across the datasets were not normally distributed, a Kruskal-Wallis test was used to assess the number of breakpoints in the three protein datasets. All pairwise comparisons were performed using Tukey’s HSD test with family-wise error correction for multiple comparisons. All statistical analysis was performed using the SciPy stats module, version 1.16.3 [60].

### Preliminary linear regression model

A preliminary linear regression model was trained on the dataset of evolutionarily conserved two-domain proteins using SciKit-Learn [60], and was then applied to the dataset of two-domain proteins unique to helminths and species-specific proteins. The average read count in the first domain was used as a predictor variable for the average read count in the second domain. Residuals, *z*-scores, and *p-*values with a Benjamini-Hochberg correction for each transcript in the helminth-only and species-specific two-domain protein datasets can be found in Supplementary Tables S4 and S5.

### *De novo* transcriptome assembly

To further investigate the authenticity of the publicly available predicted proteomes on WormBase Parasite, we performed *de novo* transcriptome assembly for all species in our dataset. The FASTQ files with adapters removed for species in our dataset were assembled into *de novo* transcriptomes using SPAdes version 4.2.0 [61]. Where more than one dataset was present for a given species, we elected to use the oldest life cycle stage. The newly generated transcriptomes were then turned into searchable BLAST+ databases [62]. BLASTn searches were performed for the two-domain architectures from the helminth-specific and species-specific architectures from these organisms with an E value threshold of 1E-6 and percent identity threshold of 95%. BLAST ‘hits’ shorter than 50% of the query protein were also removed. To verify that the remaining ‘hits’ were representative of predicted two-domain proteins, TransDecoder version 5.7.1 [63] was run on the contigs containing the ‘hits’, and PfamScan version 1.6 [64] was used to predict the domains of these proteins. The BLASTn ‘hits’ were cross referenced with the PfamScan results to ensure that the architecture of the ‘hit’ was identical to the architecture of the query protein.

### Comparing transcriptome assembly to other methods of detecting authentic annotations

The results obtained from newly generated transcriptomes were then compared to the other features investigated (interdomain length, continuity of interdomain coverage, breakpoints, preliminary linear regression model) to assess how well these features were able to predict whether a protein had transcriptomic support. The dataset of helminth- and species-specific two-domain proteins from the five species used for transcriptome generation was utilized here. Eight possible predictor variables were tested, including short interdomain lengths (< 50, < 100, and < 200 base pairs), the ratio of maximum to minimum reads spanning positions within the interdomain region (> 0.1, > 0.2, > 0.3), and having zero or fewer than one breakpoint.

Each predictor variable was assessed individually: a confusion matrix was generated, and accuracy, precision, recall (sensitivity), and F1 score were calculated to assess predictive performance. A chi-squared test of independence was used to evaluate the statistical association between each predictor and the transcriptomic evidence outcome. Additionally, a univariate logistic regression model was fitted to each predictor variable to estimate the strength of association, expressed as odds ratios (OR) with corresponding p-values.

## Results and discussion

### Existing helminth proteomes contain unusual domain architectures that are not supported by RNA-seq

The International Helminth Genomes Consortium previously identified 14,596 combinations of domains unique to helminth taxa [1]. Similarly, in our previous work investigating the absence of COX subunits and assembly factors in helminths, we identified many proteins related to COX in helminths with different domain architectures when compared to the *C. elegans* versions of these proteins. 440 identified assembly factors from 155 helminth proteomes were found to possess not only the expected COX domains present in the canonical forms of these proteins, but also additional domains unrelated to COX assembly (see Fig. 1B Supplemental Table S8). One possibility is that these proteins could be the result of gene fusions, as novel domain combinations have been described in helminths [1].

As an initial motivation for this work, we were particularly interested in protein architectures unique to helminths that involve CtaG_Cox11, the functional domain from Cox-11 (Fig. 1C), as we recently reported novel *COX11* mutations in a case of inherited mitochondrial disease [65]. Cox-11 localizes to the inner mitochondrial membrane [66, 67], and is involved in the delivery of copper ions to an assembling COX holoenzyme [68–70]. In *C. elegans*, Cox-11 has one Pfam domain, CtaG_Cox11 (PF04442). We identified 15 architectures in other helminths that contain not only the Cox-11 domain, but also additional domains not present in the *C. elegans* Cox-11 ortholog (Fig. 1C). Surprisingly, none of these additional domains share either their function or their subcellular localization with the CtaG_Cox11 domain, nor do they have any relation to COX assembly. In addition, based on InterPro and Pfam databases, none of the Cox-11 domain combinations were observed in proteomes outside of helminth taxa. For a more detailed list of architectures involving CtaG_Cox11 domains, as well as multidomain architectures identified for other COX-related proteins, please refer to Supplemental Table S8.

**Fig. 2:**
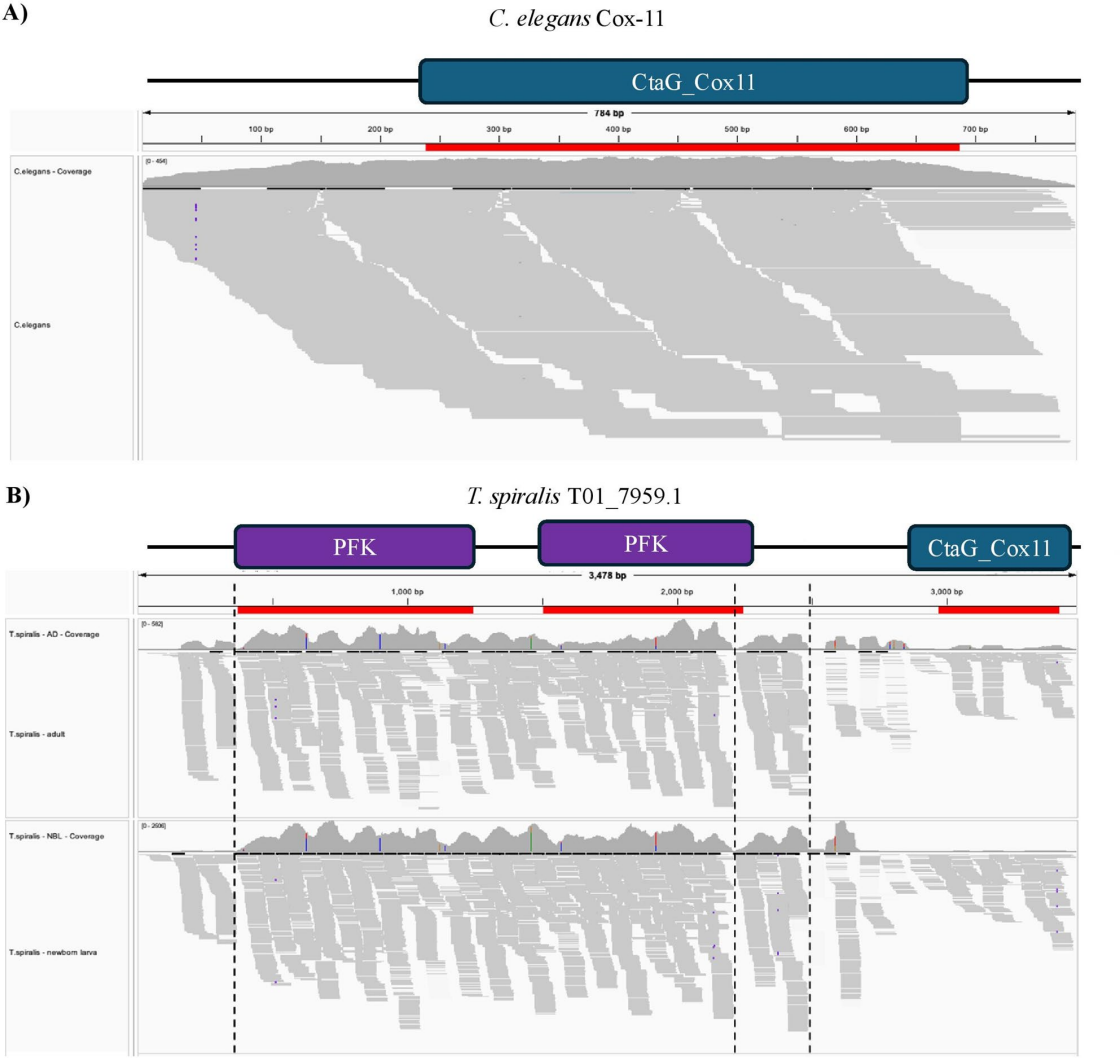
RNA-seq read coverage for two helminth genes encoding CtaG_Cox11 domain-containing proteins. Domains identified by PfamScan are shown above the coverage plots. **A** Transcript JC8.5a in *C. elegans*. This transcript and a splice variant, JC8.5b, are the only two proteins in *C. elegans* that contain the CtaG_Cox11 domain and have been annotated as Cox-11 proteins in WormBase Parasite. **B** Transcript T01_7959.1 from *T. spiralis*, in both newborn larva (NBL) and adult (AD) life cycle stages. This transcript and its four variants are the only instances of the CtaG_Cox11 domain in the *T. spiralis* proteome; all contain two copies of the PFK domain. The dotted lines indicate breakpoints where no reads span both sides of the breakpoint location

Before planning wet lab experiments to investigate the potential functionality of some of these apparent multidomain Cox-11 proteins, we explored a selection of previously published helminth RNA-seq data available on the Sequence Read Archive [33] to determine whether these multidomain fusions are supported by transcriptomic data (see Supplemental Table S9). We initially focussed on a putative Cox-11 protein we identified in *Trichinella spiralis*. This protein contains the expected CtaG_Cox11 domain, but additionally has two PFK domains, which are the functional domains found in phosphofructokinase [13], a cytosolic enzyme involved in glycolysis [66]. Notably, PFK domain functions in a different pathway and is found in a different cellular compartment from CtaG_Cox11. As shown in RNA-seq read coverage plots (Fig. 2), the single-domain Cox-11 gene from *C. elegans* (Fig. 2A) had even coverage along the full-length of the gene and encoded protein sequence. In contrast, the putative multi-domain fusion protein in *T. spiralis* showed significant variation in expression levels, including regions of high coverage and segments where coverage levels drop to zero (Fig. 2B); these patterns were observed in RNA-seq data from both *T. spiralis* newborn larva and adult life cycle stages. A clear breakpoint in RNA-seq read coverage exists between the second PFK domain and the CtaG_Cox11 domain, suggesting that this gene model has incorrectly fused two separate transcripts, and that this may be a result of either sequencing or annotation error.

### Constructing a dataset of apparent fusion genes in helminth proteomes

**Fig. 3:**
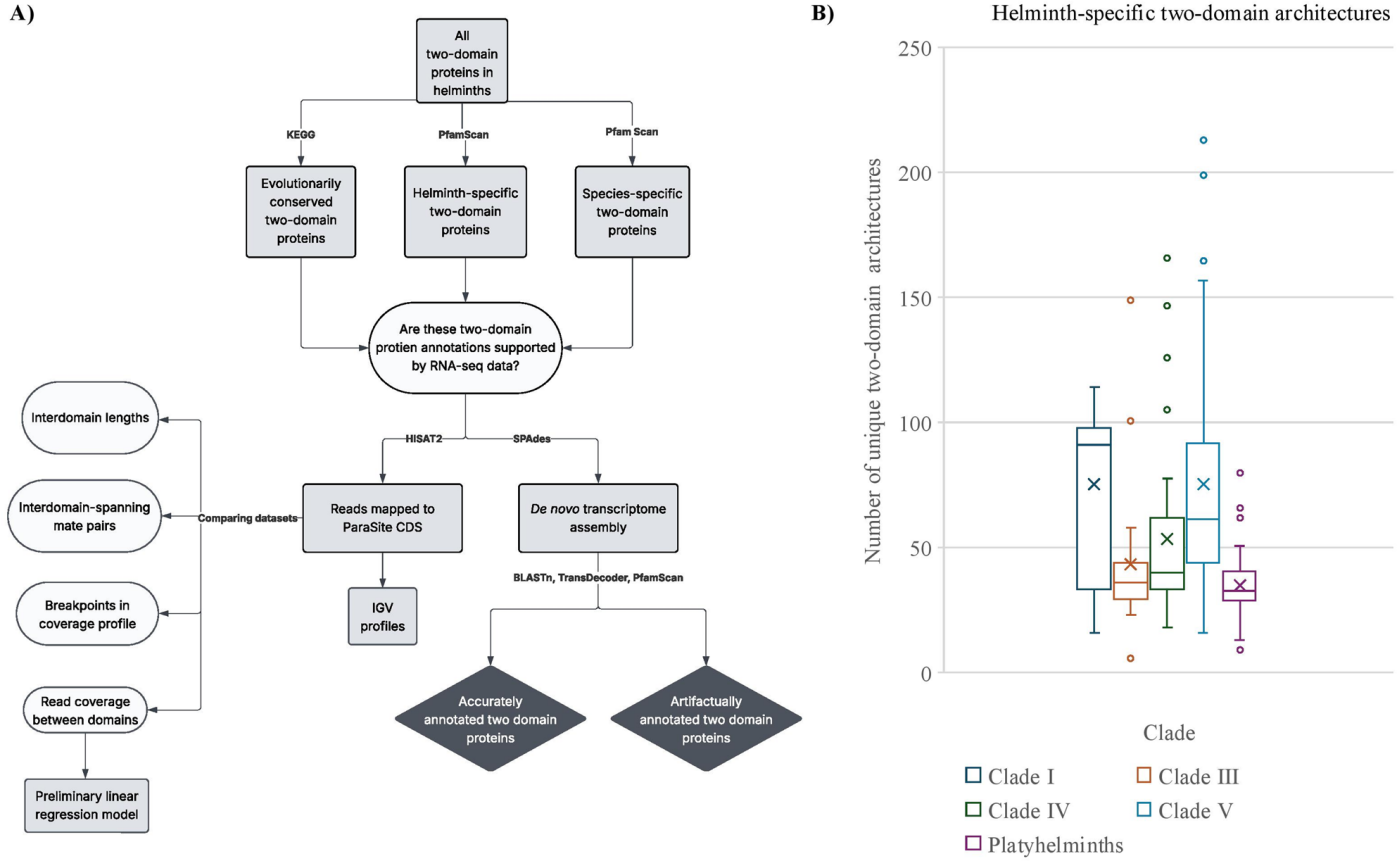
Identifying two-domain protein architectures unique to helminth taxa, and a comparison set of high-quality two-domain architectures conserved across helminth and non-helminth species. **A** Bioinformatic workflow for constructing datasets. The predicted proteomes for 155 species of helminth were retrieved from WormBase ParaSite. **B** Number of helminth-specific two-domain proteins found in each clade. After identifying all two-domain proteins found in 155 species of helminth, InterPro and PfamScan were used to identify a set of architectures found only in helminth taxa. The distribution of helminth-specific architecture counts per species within each clade is shown as a boxplot, with the X indicating the mean number of architectures per species for that clade

To analyze the RNA-seq coverage patterns of helminth gene fusions on a larger scale, we began by constructing a dataset of all two-domain architectures found in the proteomes of 155 helminth species (see Fig. 3A for methods summary). In total, we identified 20,313 architectures in these species that are comprised of two domains (see Supplemental Table S2. Using the InterPro database [13], we determined that 10,297 (51%) of these two-domain architectures are exclusive to one or more helminth species and absent outside of helminth phyla (Fig. 3B, see Supplemental Table S3 for a full list of two-domain architectures unique to helminths). We then further subdivided the set of two-domain architectures into those found in multiple species of helminth (*helminth-specific* protein dataset), and architectures found only in a single species of helminth (*species-specific* protein dataset). For each species (*n* = 155), we quantified the total number of proteins unique to helminths in that proteome. Species were then grouped by clade according to the phylogenetic tree shown in Fig. 1B. For each clade, we calculated the mean number of proteins unique to helminths by averaging the species-level counts within that clade. As shown in Fig. 3B, two-domain architectures unique to helminths are distributed across all major helminth clades with an average of 76, 43, 54, 75, in the clade I, III, IV, V nematodes respectively, and an average of 35 in the platyhelminths.

As a comparison dataset of genuine fusion genes, we constructed a set of high-quality, evolutionarily conserved two-domain proteins found in both helminth and non-helminth species. We identified 400 metabolic proteins from KEGG [59] that contain two-domains in *C. elegans*, and for which there are also more than 1,000 proteins with identical architectures in InterPro [13]; see Supplemental Table S6. These two-domain proteins are much less likely to be the result of sequencing or annotation artifacts as they are found in many well-studied organisms, allowing them to serve as a comparator group for assessing the authenticity of helminth- and species-specific multi-domain proteins.

### Helminth fusion genes have unusual sequence features and transcriptional behaviour compared to conserved fusion genes

**Fig. 4:**
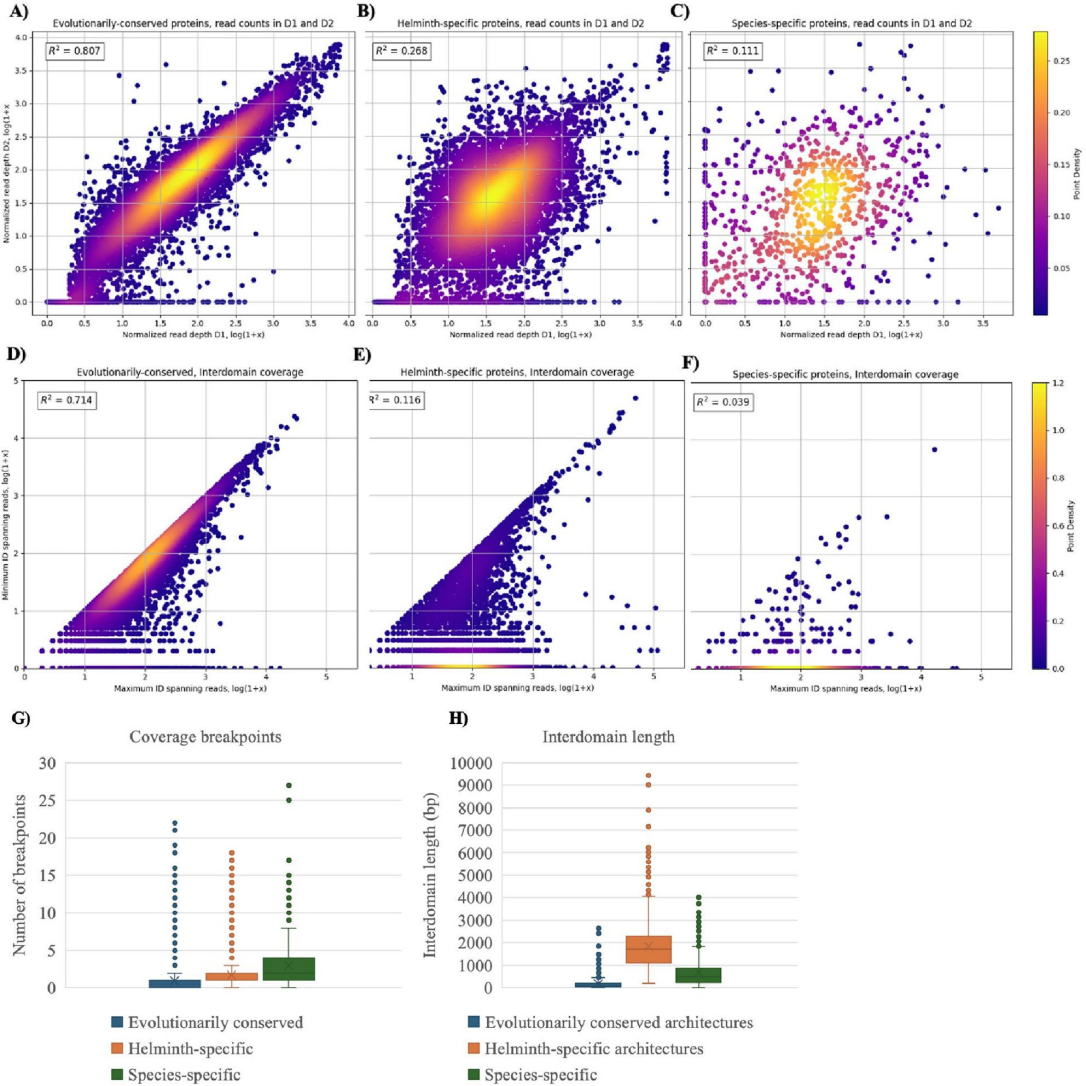
Comparing the features of evolutionarily conserved two-domain proteins to those unique to helminths. **A**-**C** Scatterplots showing correlations between mean read depths between first and second domains. PfamScan was used to annotate helminth proteomes and to predict the locations of the functional domains found within each protein. The mean read counts for the first domain (D1) and second domain (D2) were extracted from RNA-seq .bam files. Read counts were normalized to the length of the region and transformed by log(1 + x). **D**-**F** Coverage of continuity across the interdomain regions. The number of reads spanning each position in the interdomain region was determined for each protein in the datasets of two-domain proteins. The maximum and minimum number of reads were transformed by log(1 + x). **G** The number of breakpoints was counted for each protein with more than 20 reads overall. Breakpoints were defined as locations where the ratio of the average number of reads spanning a breakpoint and the total depth at those positions was less than 0.1. **H** After identifying the location of the domains of interest, the lengths of the interdomain regions were calculated for each architecture. The distribution of these lengths for each dataset is shown in a boxplot

We obtained published RNA-seq datasets from the NCBI Sequence Read Archive [33] for 20 species of helminth, as well as *C. elegans*. We recognize that this approach only represents a fraction of the available helminth transcriptomes and results in a dataset dominated by *Trichinella* species. However, our aim with this work was not to exhaustively document every potential gene fusion artifact across all helminth species with available sequencing data, but rather, to highlight the issue of potential annotation errors, providing a framework for experts in helminth assembly and annotation to revisit existing datasets. In the predicted proteomes of these species, we found 7,144 proteins with architectures found only in helminths (see Supplemental Table S4, 855 proteins with species-specific architectures (Supplemental Table S5) and 7,955 proteins (Supplemental Table S7) with highly conserved architectures. For these proteins, we compared the expression level (average normalized read count) for each of the two domains (see Fig. 1A). We hypothesized that genuine fusion genes should have similar expression levels for both domains, whereas this would not necessarily be the case for artifactual fusion genes since their two domains might be encoded by separate transcripts. Consistent with this hypothesis, the expression levels of both domains from evolutionarily conserved fusion genes were highly correlated (R^2^ = 0.807, *n* = 7,773, Fig. 4A). In contrast, expression levels of both domains from helminth-specific proteins were not highly correlated (R^2^ = 0.268, *n* = 7,144, Fig. 4B). Furthermore, the domains of species-specific fusion genes had even lower correlation (R^2^ = 0.111, *n* = 914, Fig. 4C). The difference in correlation between the conserved fusion genes and the helminth-specific and species-specific fusion genes was highly significant according to Z-tests (*p* = 0 for both comparisons).

As a further means to comparing the evolutionarily conserved, helminth-specific, and species-specific datasets of two-domain proteins, we were interested in assessing the continuity of the RNA-seq read coverage profiles, which was achieved in two different ways. First, we looked at the interdomain region specifically by counting the number of reads spanning each position within the region. We theorized that for accurately annotated proteins, the ratio between the maximum and minimum number of reads here would be close to 1 and would be highly correlated on a scatterplot. Consistent with this hypothesis, the evolutionarily conserved protein dataset was strongly correlated (R^2^ = 0.714, Fig. 4D), and 17% of genes had a minimum read count of 0. The helminth specific proteins (R^2^ = 0.116, 67% of genes where minimum read count = 0, Fig. 4E) and species-specific proteins (R^2^ = 0.039, 85% of genes where minimum read count = 0, Fig. 4F) had much lower degrees of correlation. The difference in correlation between the conserved two-domain genes and the helminth-specific and species-specific genes was highly significant according to Z-tests (*p* = 0 for both comparisons). There was additionally a weakly significant difference between the helminth- and species-specific datasets (*p* = 0.02759).

As a second method to assess continuity of coverage, we investigated the RNA-seq read coverage profiles, looking for locations across the protein where coverage levels drop close to zero, such as the breakpoints indicated in Fig. 2B. We defined breakpoints as positions with a normalized read coverage of below 0.1, representing a significant drop in coverage across a junction while allowing for some background noise. The number of breakpoints was counted for all genes in our dataset with > = 20 mapped reads (see Fig. 4G). The evolutionarily conserved fusions had significantly fewer breakpoints (mean = 0.96, *n* = 7663) than both the helminth-specific and species-specific datasets; the species-specific two-domain proteins had significantly more breakpoints (mean = 2.98, *n* = 825) than the helminth-specific proteins (mean = 1.74, *n* = 7084).

Next, we hypothesized that artifactual fusions may deviate from true fusion genes in the length of the gap separating both domains. We therefore measured the interdomain distances of proteins in our dataset (Fig. 4H). Indeed, evolutionarily conserved proteins were found to have shorter interdomain lengths (mean = 180 bp) than two-domain proteins unique to helminths (mean = 1851 bp) and the helminth species-specific proteins (mean = 691 bp). The interdomain lengths of the helminth-specific two domain proteins were significantly longer than those of the other two datasets (*p* < 0.001); the interdomain lengths of the species-specific proteins were also significantly longer (*p* < 0.001) than those of the evolutionarily conserved proteins. Taken together, our analyses reveal striking differences between the evolutionarily conserved two-domain proteins and the helminth- and species-specific two-domain proteins, which could represent surprisingly widespread misannotations with respect to putative fusion proteins in the currently available helminth genome databases.

We also considered whether the RNAseq expression profiles that resemble gene fusion artifacts could arise from pre-mRNA polycistronic transcripts prior to undergoing spliced leader (SL) trans-splicing, which is an interesting feature of some helminth transcripts. SL trans-splicing adds a spliced-leader exon from an independently transcribed SL RNA, to the 5′ end of a pre-mRNA transcript [71], which might also explain the observed internal breakpoints. To assess the possibility of SL trans-splicing, we compared our list of helminth-specific two-domain proteins to the full registry of platyhelminth SL trans-splicing acceptor sites provided in Calvelo et al. (2025) [71]. We looked at the overlapping species between our dataset and Calvelo et al.’s, (*Echinococcus multilocularis*) and found no overlap between our candidate fusion-artifact genes and the genes supported by SL-bearing reads in their dataset. Although this test is limited to one species, the complete absence of overlap suggests that SL trans-splicing is unlikely to account for the majority of cases we identify. Moreover, even if a subset were influenced by trans-splicing, this would still highlight a broader issue: current annotation pipelines may not reliably distinguish true coding sequences from chimeric or polycistronic transcriptional units in helminth genomes.

### Visualization of example fusion gene artifacts

In the scatterplots shown in Fig. 4B and C, data points that are far from the y = x line represent proteins whose domains have the largest differences in expression level, indicative of gene prediction artifacts. To identify these quantitatively, we built a linear regression model of D1 versus D2 expression level for evolutionarily conserved two-domain proteins and then applied this model to score the helminth-specific and species-specific datasets (see Supplementary Figures S4 and S5 for residual scores). Based on the residuals, we were able to score all two domain proteins unique to helminths based on their deviation from expected behaviour, facilitating identification of likely gene prediction artifacts.

**Fig. 5:**
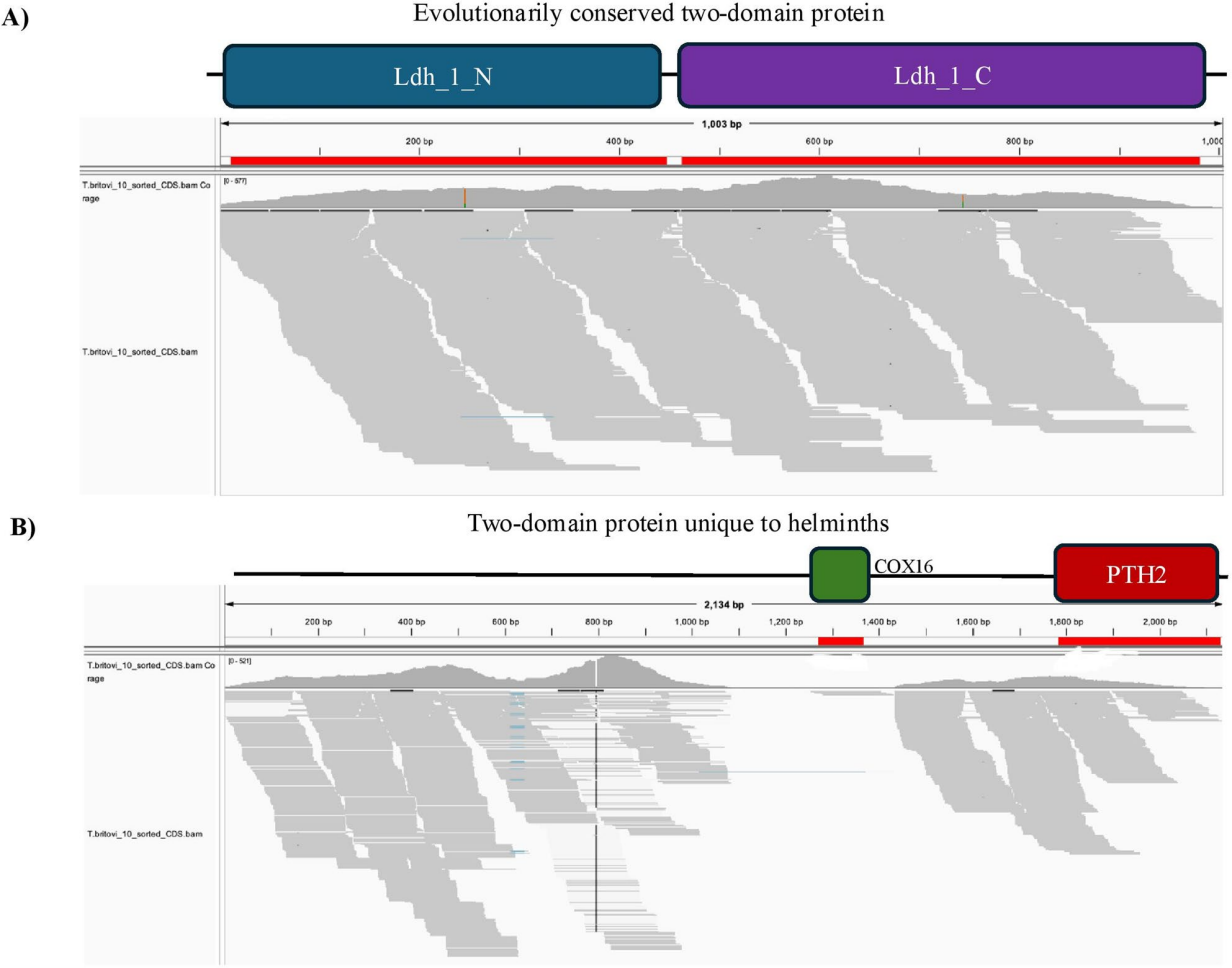
Sample two-domain proteins from the evolutionarily conserved and helminth-only datasets. **A** Transcript T03_15976.1 from *T. britovi* mid-stage larva. PfamScan identifies two domains in this protein, Ldh_1_N and Ldh_1_C, separated by an interdomain region of 14 bp. **B** Transcript T03_10406.1 from *T. britovi* mid-stage larva. This protein contains a COX16 domain combined with a PTH2 domain, separated by an interdomain region of 416 bp

To further visualize the differences between our evolutionarily conserved proteins and those unique to helminths, we examined the RNA-seq coverage plots for a subset of proteins using IGV [57]. Figure 5A provides a sample RNA-seq profile of a randomly selected, evolutionarily conserved two-domain protein: clearly, there are no visible breakpoints in the expression profile, nor are there any places in the transcript where the number of reads drops to zero. The interdomain length is very short, at only 14 bp. By contrast, Fig. 5B displays a sample expression profile from the helminth-specific two-domain architectures, containing a COX-related domain (COX16) with an unrelated domain (PTH2) and featuring a longer interdomain region (416 bp) and a clear breakpoint in the coverage profiles where there are no reads mapping. Interestingly, Pfam does not identify any domains in the first segment of the protein, although this is the region with the highest expression levels. Although the breakpoint does not occur between the two predicted domains, this protein has clearly been incorrectly annotated.

We additionally looked at the IGV profiles for 20 of our helminth-specific two-domain proteins, which we determined to be artifactually annotated as having two domains (see Supplemental Figure S1). These proteins were identified based on having the lowest degree of correlation between their normalized read counts in the two domains. The interdomain lengths of these proteins ranged from 35 to 800 bp and all transcripts had at least one obvious breakpoint in their expression profiles. These proteins had highly variable expression levels, like the profile displayed in Fig. 5B.

### De Novo transcriptome assembly distinguishes between authentic and erroneous two-domain proteins

Next, we reasoned that authentic two-domain proteins should be far more likely to be validated by assembled transcripts than artifactual fusion genes. To investigate this, we utilized the same RNA-seq datasets to generate *de novo* transcriptomes. After performing *de novo* transcriptome assembly using SPAdes [61], BLASTn was used to search the assembled transcripts for the coding sequences of the evolutionarily conserved, helminth-specific, and species-specific two-domain proteins present in these organisms as queries. *E*-value and percent identity thresholds were set at 1E-6 and 95% respectively; additionally, only BLASTn ‘hits’ that were greater than 50% the length of the query protein were investigated to maximize the chance of finding genuine two-domain proteins in the new transcriptome. Once contigs containing sequence ‘hits’ were identified, open reading frames were predicted, and PfamScan was then used to further verify whether the predicted proteins contained the expected two domains. A query two-domain protein with a BLASTn ‘hit’ that met these criteria, and for which the ‘hit’ contained the appropriate domains, was considered as having transcriptomic support.

**Fig. 6:**
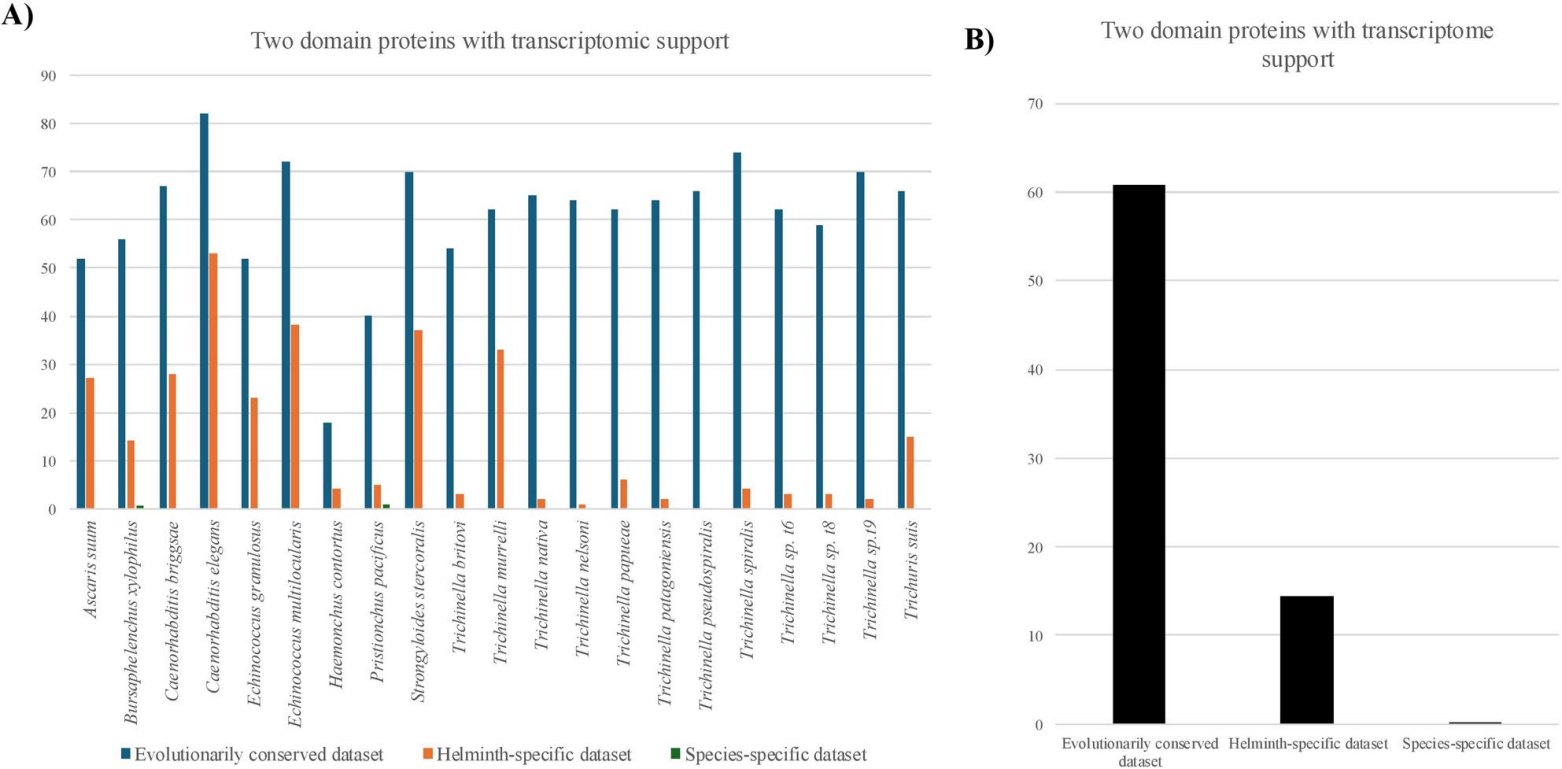
Two-domain proteins in helminths with transcriptome support. **A** *De novo* transcriptomes were generated from RNA-seq data for species of helminth used in this project. The newly generated transcriptomes were searched using BLASTn for contigs that could encode for two-domain proteins; open reading frames were predicted from contigs identified as BLAST ‘hits’; PfamScan was used to verify the presence of the two domains. **B** Average number of two-domain proteins per species with transcriptome support

Of the BLAST ‘hits’ of adequate length, 61% of the two-domain evolutionarily conserved proteins were identified in the transcriptome, whereas only 14% of proteins in the helminth-specific dataset were supported. From the two-domain proteins in the helminth-specific dataset which had transcriptomic support, we identified 43 non-redundant architectures (see Supplemental Table S9). Strikingly, only 0.1% of the species-specific two-domain proteins had BLASTn ‘hits’ in the transcriptome (Fig. 6).

We also compared the transcriptomic results to the predictor variables investigated earlier: interdomain length, the ratio of maximum to minimum number of reads spanning the interdomain regions, and the number of breakpoints in the expression profiles. Predictive performance was tested for each of these variables independently using a confusion matrix and chi-squared test of independence. We first tested interdomain length to determine whether shorter lengths were predictive of transcriptomic support. Interdomain lengths of less than 50, 100, or 200 bases were assessed individually. A length of less than 50 bases provided the most accurate predictive performance (accuracy = 0.67, F1 = 0.35, odds ratio = 2.04, precision = 0.44 recall = 0.29, *p* < 0.00000), although longer thresholds (< 100 and < 200 bp) had higher F1 scores and stronger odds ratios. We also evaluated the ratio of the maximum and minimum number of reads spanning positions in the interdomain region. Having a ratio greater than 0.1 (accuracy = 0.73, F1 = 0.68, odds ratio = 17.94, precision = 0.54, recall = 0.90, *p* < 0.00000) and greater than 0.2 (accuracy = 0.75, F1 = 0.68, odds ratio = 13.95, precision = 0.56, recall = 0.86, *p* < 0.00000) were strongly predictive of having transcriptomic support, with moderate predictive power overall. A ratio greater than 0.3 was also predictive, though less strongly (accuracy = 0.75, F1 = 0.66, odds ratio = 10.27, precision = 0.56, recall = 0.80, *p* < 0.00000). We performed a similar analysis for breakpoints in expression profiles: having no breakpoints was associated with increased odds of transcriptomic support (accuracy = 0.70, F1 = 0.57, odds ratio = 4.82, precision = 0.51, recall = 0.64, *p* < 0.00000).

## Conclusions

Based on the analyses presented here, the expected characteristics of a correctly annotated two-domain protein are: (1) relatively even read coverage across the length of the protein; (2) short interdomain length; (3) consistent coverage across the interdomain region with the ratio of the maximum and minimum number of position spanning reads being close to 1; and (4) no breakpoints in the expression profile. Collectively, these features could be used to assess whether a novel two-domain architecture has been correctly annotated; in the absence of transcriptomic data, the interdomain length between predicted domains could be used as a preliminary screen for putative annotation artifacts. Although we only considered two-domain architectures, there could also be a significant number of artifacts among transcripts with larger numbers of domains, as seen with the transcript shown in Fig. 2B, which is annotated as containing two PFK domains and a CtaG_Cox11 domain.

Our analyses of a selection of publicly available RNAseq data clearly reveal unexpected anomalies in genome assembly annotations and underline the challenges associated with both current technical and informatic approaches and the need for more precise curation of the resultant databases. The data presented suggest that the list of two-domain architectures unique to helminths contains a significant proportion of artifacts, perhaps as high as 86%, based on our analyses. Proposed novel architectures should therefore be investigated on a case-by-case basis, and new architectures found in multiple species should be considered questionable until additional data can confirm their accuracy. Having accurately annotated helminth proteomes is essential to understanding helminth metabolism, and its divergence from the hosts, which is an important first step towards the development of novel anthelminthic agents.

## Supplementary Information

Supplementary Material 1.

Supplementary Material 2.

## Data Availability

All RNA-seq datasets utilized in this project are publicly available on the NCBI Sequence Read Archive. Reference genomes used are available on WormBase ParaSite. All relevant material has been included in Supplemental Data files.

## Abbreviations

COX: Cytochrome c oxidase

## References

[1] Coghlan A, Tyagi R, Cotton JA, Holroyd N, Rosa BA, Tsai IJ, et al. Comparative genomics of the major parasitic worms. Nat Genet. 2019;51(1):163–74. 30397333 10.1038/s41588-018-0262-1 PMC6349046

[2] Lustigman S, Prichard RK, Gazzinelli A, Grant WN, Boatin BA, McCarthy JS et al. CD <>Mackenzie editor 2012 A research agenda for helminth diseases of humans: the problem of helminthiases. PLoS Negl Trop Dis 6 4 e1582. 22545164 10.1371/journal.pntd.0001582 PMC3335854

[3] McCarter JP. Genomic filtering: an approach to discovering novel antiparasitics. Trends Parasitol. 2004;20(10):462–8. 15363439 10.1016/j.pt.2004.07.008

[4] Williams SA. The filarial genome Project, Johnston DA, the schistosome genome Project. Helminth genome analysis: the current status of the filarial and schistosome genome projects. Parasitology. 1999;118(7):19–38. 10.1017/s0031182099004473 10466135

[5] Brindley PJ, Mitreva M, Ghedin E, Lustigman S. Helminth genomics: The implications for human health. Knight M, editor. PLoS Negl Trop Dis. 2009;3(10):e538. 10.1371/journal.pntd.0000538 PMC2757907 19855829

[6] Bockarie MJ, De Souza DK, Ansumana R, Ladzekpo D, Ramaiah KD, Karutu C, et al. The changing funding landscape for infectious disease research and control: implications for resource-limited countries. Int J Infect Dis. 2025;154:107868. 40032134 10.1016/j.ijid.2025.107868

[7] Anderson RM, Cano J, Hollingsworth TD, Deribe-Kassaye K, Zouré HGM, Kello AB, et al. Responding to the cuts in UK AID to neglected tropical diseases control programmes in Africa. Trans R Soc Trop Med Hyg. 2023;117(3):237–9. 36416069 10.1093/trstmh/trac109 PMC9977241

[8] Howe KL, Bolt BJ, Shafie M, Kersey P, Berriman M. WormBase ParaSite – a comprehensive resource for helminth genomics. Mol Biochem Parasitol. 2017;1:215:2–10. 10.1016/j.molbiopara.2016.11.005 PMC5486357 27899279

[9] Harris TW, Lee R, Schwarz E, Bradnam K, Lawson D, Chen W, et al. WormBase: a cross-species database for comparative genomics. Nucleic Acids Res. 2003;31(1):133–7. 12519966 10.1093/nar/gkg053 PMC165500

[10] Doyle SR. Improving helminth genome resources in the post-genomic era. Trends Parasitol. 2022;38(10):831–40. 35810065 10.1016/j.pt.2022.06.002

[11] Shanmugam D, Ralph SA, Carmona SJ, Crowther GJ, Roos DS, Agüero F. Integrating and mining helminth genomes to discover and prioritize novel therapeutic targets. In: Caffrey CR, editor. Parasitic Helminths. 1st ed. Wiley; 2012. pp. 43–59. Available from: https://onlinelibrary.wiley.com/doi/10.1002/9783527652969.ch3. Cited 14 Apr 2025.

[12] Hagen J, Lee EF, Fairlie WD, Kalinna BH. Functional genomics approaches in parasitic helminths. Parasite Immunol. 2012;34(2–3):163–82. 21711361 10.1111/j.1365-3024.2011.01306.x

[13] Blum M, Andreeva A, Florentino LC, Chuguransky SR, Grego T, Hobbs E, et al. InterPro: the protein sequence classification resource in 2025. Nucleic Acids Res. 2025;53(D1):D444–56. 39565202 10.1093/nar/gkae1082 PMC11701551

[14] Ashburner M, Ball CA, Blake JA, Botstein D, Butler H, Cherry JM, et al. Gene ontology: tool for the unification of biology. Nat Genet. 2000;25(1):25–9. 10802651 10.1038/75556 PMC3037419

[15] The Gene Ontology Consortium. The gene ontology resource: enriching a gold mine. Nucleic Acids Res. 2021;49(D1):D325–34. 33290552 10.1093/nar/gkaa1113 PMC7779012

[16] Palevich N, Britton C, Kamenetzky L, Mitreva M, De Moraes Mourão M, Bennuru S, et al. Tackling hypotheticals in helminth genomes. Trends Parasitol. 2018;34(3):179–83. 29249363 10.1016/j.pt.2017.11.007 PMC11021132

[17] Murzin AG, Brenner SE, Hubbard T, Chothia C. SCOP: A structural classification of proteins database for the investigation of sequences and structures. J Mol Biol. 1995;247(4):536–40. 7723011 10.1006/jmbi.1995.0159

[18] Dawson N, Sillitoe I, Marsden RL, Orengo CA. The classification of protein domains. Bioinformatics. 2017;137:64. 10.1007/978-1-4939-6622-6_7 27896721

[19] Elofsson A, Sonnhammer ELL. A comparison of sequence and structure protein domain families as a basis for structural genomics. Bioinformatics. 1999;15(6):480–500. 10383473 10.1093/bioinformatics/15.6.480

[20] Ekman D, Björklund ÅK, Frey-Skött J, Elofsson A. Multi-domain proteins in the three kingdoms of life: orphan domains and other unassigned regions. J Mol Biol. 2005;348(1):231–43. 15808866 10.1016/j.jmb.2005.02.007

[21] Forslund K, Sonnhammer ELL. Evolution of protein domain architectures. In: Anisimova M, editor. Evolutionary Genomics: Statistical and Computational Methods. Totowa, NJ: Humana Press; 2012;2:187–216. Available from: 10.1007/978-1-61779-585-5_8. 10.1007/978-1-61779-585-5_8 22399460

[22] Apic G, Gough J, Teichmann SA. Domain combinations in archaeubacterial and eukaryotic proteomes. von Heijne, G., editor. Journal of molecular biology. 2001;310(2):311–25. 10.1006/jmbi.2001.4776 11428892

[23] Han JH, Batey S, Nickson AA, Teichmann SA, Clarke J. The folding and evolution of multidomain proteins. Nat Rev Mol Cell Biol. 2007;8(4):319–30. 17356578 10.1038/nrm2144

[24] Björklund ÅK, Ekman D, Light S, Frey-Skött J, Elofsson A. Domain rearrangements in protein evolution. J Mol Biol. 2005;353(4):911–23. 16198373 10.1016/j.jmb.2005.08.067

[25] Bashton M, Chothia C. The generation of new protein functions by the combination of domains. Struct (London). 2007;15(1):85–99. 10.1016/j.str.2006.11.009 17223535

[26] Collington E, Lobb B, Mazen NA, Doxey AC, Glerum DM. Phylogenomic analysis of 155 helminth species reveals widespread absence of oxygen metabolic capacity. Gossmann T, editor. Genome Biology and Evolution. 2023;15(8):evad135. 10.1093/gbe/evad135 PMC10400150 37481257

[27] Pasek S, Risler JL, Brézellec P. Gene fusion/fission is a major contributor to evolution of multi-domain bacterial proteins. Bioinformatics. 2006;22(12):1418–23. 16601004 10.1093/bioinformatics/btl135

[28] Marsh JA, Teichmann SA. How do proteins gain new domains? GENOME BIOL. 2010;11(7):126–126. 20630117 10.1186/gb-2010-11-7-126 PMC2926777

[29] Orengo CA, Thornton JM. Protein families and their evolution - A structural perspective. Annu Rev Biochem. 2005;74(1):867–900. 15954844 10.1146/annurev.biochem.74.082803.133029

[30] Lobb B, Doxey AC. Novel function discovery through sequence and structural data mining. Curr Opin Struct Biol. 2016;38:53–61. 10.1016/j.sbi.2016.05.017 27289211

[31] Thorn CS, Maness RW, Hulke JM, Delmore KE, Criscione CD. Population genomics of helminth parasites. J Helminthol. 2023;97:e29. 36927601 10.1017/S0022149X23000123

[32] Castañeda S, Ramírez JD. From genes to worms: A deep dive into helminth omics. In: Ramírez González JD, editor. Recent Advances in Parasitomics. Cham: Springer Nature Switzerland; 2025. pp. 207–46. Available from: https://link.springer.com/10.1007/978-3-031-70591-5_12. Cited 24 Apr 2025.

[33] Katz K, Shutov O, Lapoint R, Kimelman M, Brister JR, O’Sullivan C. The sequence read archive: a decade more of explosive growth. Nucleic Acids Res. 2022;50(D1):D387–90. 34850094 10.1093/nar/gkab1053 PMC8728234

[34] NCBI Resource Coordinators. Database resources of the National center for biotechnology information. Nucleic Acids Res. 2016;44(D1):D7–19. 26615191 10.1093/nar/gkv1290 PMC4702911

[35] Jex AR, Liu S, Li B, Young ND, Hall RS, Li Y, et al. Ascaris suum draft genome. Nature. 2011;479(7374):529–33. 22031327 10.1038/nature10553

[36] Wu S, Gao S, Wang S, Meng J, Wickham J, Luo S, et al. A reference genome of bursaphelenchus mucronatus provides new resources for revealing its displacement by Pinewood nematode. Genes. 2020;11(5):570. 32438771 10.3390/genes11050570 PMC7288286

[37] Ceron-Noriega A, Almeida MV, Levin M, Butter F. Nematode gene annotation by machine-learning-assisted proteotranscriptomics enables proteome-wide evolutionary analysis. Genome Res. 2023;33(1):112–28. 36653121 10.1101/gr.277070.122 PMC9977148

[38] Louisiana State University. RNA-seq of C. elegans: WT. SRR. 2024. Available from: https://www.ncbi.nlm.nih.gov/sra/?term=SRR28206915.

[39] Liu S, Zhou X, Hao L, Piao X, Hou N, Chen Q. Genome-wide transcriptome analysis reveals extensive alternative splicing events in the Protoscoleces of Echinococcus granulosus and Echinococcus multilocularis. Front Microbiol. 2017;8:929. 28588571 10.3389/fmicb.2017.00929 PMC5440512

[40] Reyes-Guerrero DE, Jiménez-Jacinto V, Alonso-Morales RA, Alonso-Díaz MÁ, Maza-Lopez J, Camas-Pereyra R, et al. Assembly and analysis of Haemonchus contortus transcriptome as a tool for the knowledge of Ivermectin resistance mechanisms. Pathogens. 2023;12(3):499. 36986421 10.3390/pathogens12030499 PMC10059914

[41] Rodpai R, Sanpool O, Thanchomnang T, Laoraksawong P, Sadaow L, Boonroumkaew P et al. Exposure to dexamethasone modifies transcriptomic responses of free-living stages of Strongyloides stercoralis. Dillman AR, editor. PLoS ONE. 2021 June 28;16(6):e0253701. 10.1371/journal.pone.0253701 PMC8238218 34181669

[42] Feng Y, Liu X, Liu Y, Tang B, Bai X, Li C, et al. Comparative epigenomics reveals host diversity of the Trichinella epigenomes and their effects on differential parasitism. Front Cell Dev Biol. 2021;11:9:681839. 10.3389/fcell.2021.681839 PMC8226246 34179010

[43] Jex AR, Nejsum P, Schwarz EM, Hu L, Young ND, Hall RS, et al. Genome and transcriptome of the Porcine whipworm trichuris suis. Nat Genet. 2014;46(7):701–6. 10.1038/ng.3012 PMC4105696 24929829

[44] Andrews S, FastQC. A quality control tool for high throughput sequence data [Internet]. Babraham Bioinformatics; 2010. Available from: http://www.bioinformatics.babraham.ac.uk/projects/fastqc

[45] Bolger AM, Lohse M, Usadel B. Trimmomatic: a flexible trimmer for illumina sequence data. Bioinformatics. 2014;30(15):2114–20. 24695404 10.1093/bioinformatics/btu170 PMC4103590

[46] Kim D, Paggi JM, Park C, Bennett C, Salzberg SL. Graph-based genome alignment and genotyping with HISAT2 and HISAT-genotype. Nat Biotechnol. 2019;37(8):907–15. 31375807 10.1038/s41587-019-0201-4 PMC7605509

[47] Jex AR, Nejsum P, Schwarz EM, Hu L, Young ND, Hall RS, et al. Genome and transcriptome of the Porcine whipworm *Trichuris suis*. Nat Genet. 2014;46(7):701–6. 10.1038/ng.3012 PMC4105696 24929829

[48] Korhonen PK, Pozio E, La Rosa G, Chang BCH, Koehler AV, Hoberg EP, et al. Phylogenomic and biogeographic reconstruction of the *Trichinella* complex. Nat Commun. 2016;7(1):10513. 26830005 10.1038/ncomms10513 PMC4740406

[49] Schwarz EM, Korhonen PK, Campbell BE, Young ND, Jex AR, Jabbar A, et al. The genome and developmental transcriptome of the strongylid nematode *Haemonchus contortus*. Genome Biol. 2013;14(8):R89. 23985341 10.1186/gb-2013-14-8-r89 PMC4053716

[50] The Taenia solium Genome Consortium, Tsai IJ, Zarowiecki M, Holroyd N, Garciarrubio A, Sanchez-Flores A, et al. The genomes of four tapeworm species reveal adaptations to parasitism. Nature. 2013;496(7443):57–63. 23485966 10.1038/nature12031 PMC3964345

[51] Zheng H, Zhang W, Zhang L, Zhang Z, Li J, Lu G, et al. The genome of the hydatid tapeworm *Echinococcus granulosus*. Nat Genet. 2013;45(10):1168–75. 24013640 10.1038/ng.2757

[52] Kikuchi T, Cotton JA, Dalzell JJ, Hasegawa K, Kanzaki N, McVeigh P et al. Genomic insights into the origin of parasitism in the emerging plant pathogen *Bursaphelenchus xylophilus*. Tyler B, editor. PLoS Pathog. 2011;7(9):e1002219. 10.1371/journal.ppat.1002219 PMC3164644 21909270

[53] Wang J, Mitreva M, Berriman M, Thorne A, Magrini V, Koutsovoulos G, et al. Silencing of germline-expressed genes by DNA elimination in somatic cells. Dev Cell. 2012;23(5):1072–80. 23123092 10.1016/j.devcel.2012.09.020 PMC3620533

[54] Max-Planck Institute. Pristionchus pacificus strain:PS312. https://www.ncbi.nlm.nih.gov/bioproject/?term=PRJNA12644: BioProject; 2009.

[55] Wellcome Sanger Institute. Strongyloides stercoralis strain:PV0001. BioProject. 2014. Available from: https://www.ncbi.nlm.nih.gov/bioproject/?term=PRJEB528.

[56] Li H, Handsaker B, Wysoker A, Fennell T, Ruan J, Homer N, et al. The sequence Alignment/Map format and samtools. Bioinformatics. 2009;25(16):2078–9. 19505943 10.1093/bioinformatics/btp352 PMC2723002

[57] Robinson JT, Thorvaldsdóttir H, Winckler W, Guttman M, Lander ES, Getz G, et al. Integrative genomics viewer. Nat Biotechnol. 2011;29(1):24–6. 21221095 10.1038/nbt.1754 PMC3346182

[58] Madeira F, Park Ymi, Lee J, Buso N, Gur T, Madhusoodanan N, et al. The EMBL-EBI search and sequence analysis tools APIs in 2019. Nucleic Acids Res. 2019;47(W1):W636–41. 10.1093/nar/gkz268 PMC6602479 30976793

[59] Kanehisa M, Furumichi M, Sato Y, Ishiguro-Watanabe M, Tanabe M. KEGG: integrating viruses and cellular organisms. Nucleic Acids Res. 2021;49(D1):D545–51. 33125081 10.1093/nar/gkaa970 PMC7779016

[60] Virtanen P, Gommers R, Oliphant TE, Haberland M, Reddy T, Cournapeau D, et al. SciPy 1.0: fundamental algorithms for scientific computing in python. Nat Methods. 2020;17(3):261–72. 32015543 10.1038/s41592-019-0686-2 PMC7056644

[61] Bankevich A, Nurk S, Antipov D, Gurevich AA, Dvorkin M, Kulikov AS, et al. SPAdes: A new genome assembly algorithm and its applications to single-cell sequencing. J Comput Biol. 2012;19(5):455–77. 22506599 10.1089/cmb.2012.0021 PMC3342519

[62] Camacho C, Coulouris G, Avagyan V, Ma N, Papadopoulos J, Bealer K, et al. BLAST+: architecture and applications. BMC Bioinformatics. 2009;10(1):421. 20003500 10.1186/1471-2105-10-421 PMC2803857

[63] Grabherr MG, Haas BJ, Yassour M, Levin JZ, Thompson DA, Amit I, et al. Full-length transcriptome assembly from RNA-Seq data without a reference genome. Nat Biotechnol. 2011;29(7):644–52. 10.1038/nbt.1883 PMC3571712 21572440

[64] El-Gebali S, Mistry J, Bateman A, Eddy SR, Luciani A, Potter SC, et al. The Pfam protein families database in 2019. Nucleic Acids Res. 2019;47(D1):D427–32. 30357350 10.1093/nar/gky995 PMC6324024

[65] Caron-Godon CA, Della Vecchia S, Romano A, Doccini S, Dal Canto F, Pasquariello R, et al. Novel COX11 mutations associated with mitochondrial disorder: functional characterization in patient fibroblasts and Saccharomyces cerevisiae. IJMS. 2023;24(23):16636. 38068960 10.3390/ijms242316636 PMC10706101

[66] The UniProt Consortium, Bateman A, Martin MJ, Orchard S, Magrane M, Agivetova R, et al. UniProt: the universal protein knowledgebase in 2021. Nucleic Acids Res. 2021;49(D1):D480–9. 33237286 10.1093/nar/gkaa1100 PMC7778908

[67] Carr HS, Maxfield AB, Horng YC, Winge DR. Functional analysis of the domains in Cox11. J Biol Chem. 2005;280(24):22664–9. 10.1074/jbc.M414077200 15840584

[68] Carr HS, George GN, Winge DR. Yeast Cox11, a protein essential for cytochrome c oxidase assembly, is a Cu(I)-binding protein. J Biol Chem. 2002;277(34):31237–42. 12063264 10.1074/jbc.M204854200

[69] Hiser L, Di Valentin M, Hamer AG, Hosler JP. Cox11p is required for stable formation of the Cu band magnesium centers of cytochrome *c* oxidase. J Biol Chem. 2000;275(1):619–23. 10617659 10.1074/jbc.275.1.619

[70] Banting GS, Glerum DM. Mutational analysis of the *Saccharomyces cerevisiae* cytochrome *c* pxidase assembly protein Cox11p. Eukaryot Cell. 2006;5(3):568–78. 16524911 10.1128/EC.5.3.568-578.2006 PMC1398067

[71] Calvelo J, Musto H, Koziol U, Iriarte A. Evolution of SL-RNA genes and their splicing targets in parasitic flatworms. Ruvinsky I, editor. Molecular Biology and Evolution. 2025;42(11):msaf228. 10.1093/molbev/msaf228 PMC12582326 40985261

[72] Heger A. pysam: Python interface for the SAM/BAM format. 2019. Available from: https://github.com/pysam-developers/pysam.

