# Supplementary material for: Widespread gene fusion artifacts in helminth genome annotations
Source: BMC Genomics. 2026 Feb 4;27:253. doi: 10.1186/s12864-026-12589-y (PMC12964723; doi:10.1186/s12864-026-12589-y)
Supplement: Supplementary file 2 — Supplementary Material 2. [file 12864_2026_12589_MOESM2_ESM.docx]

**
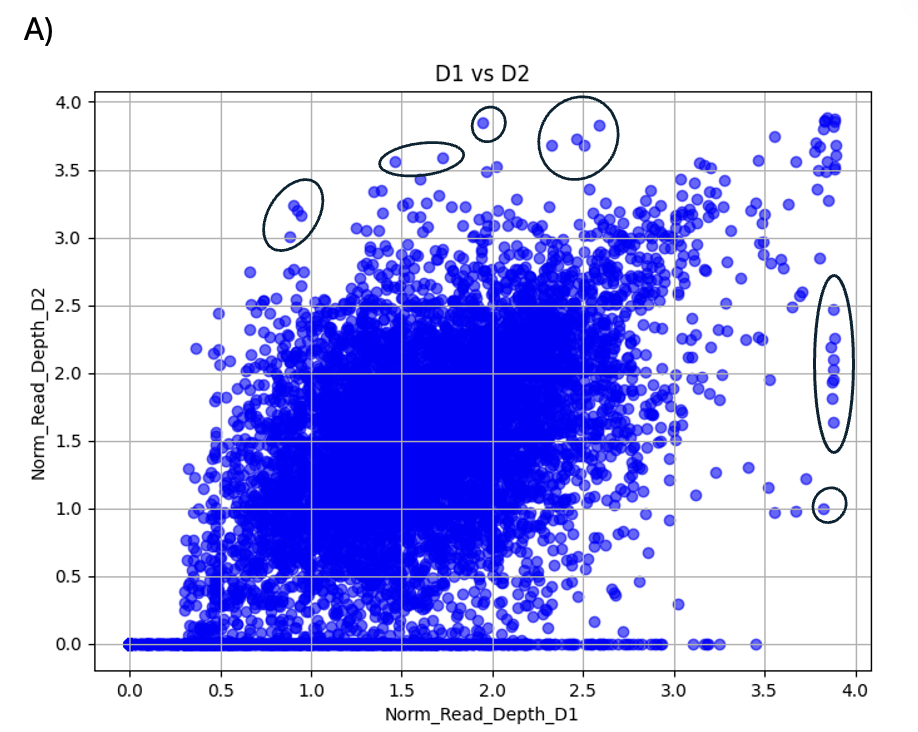
**

**
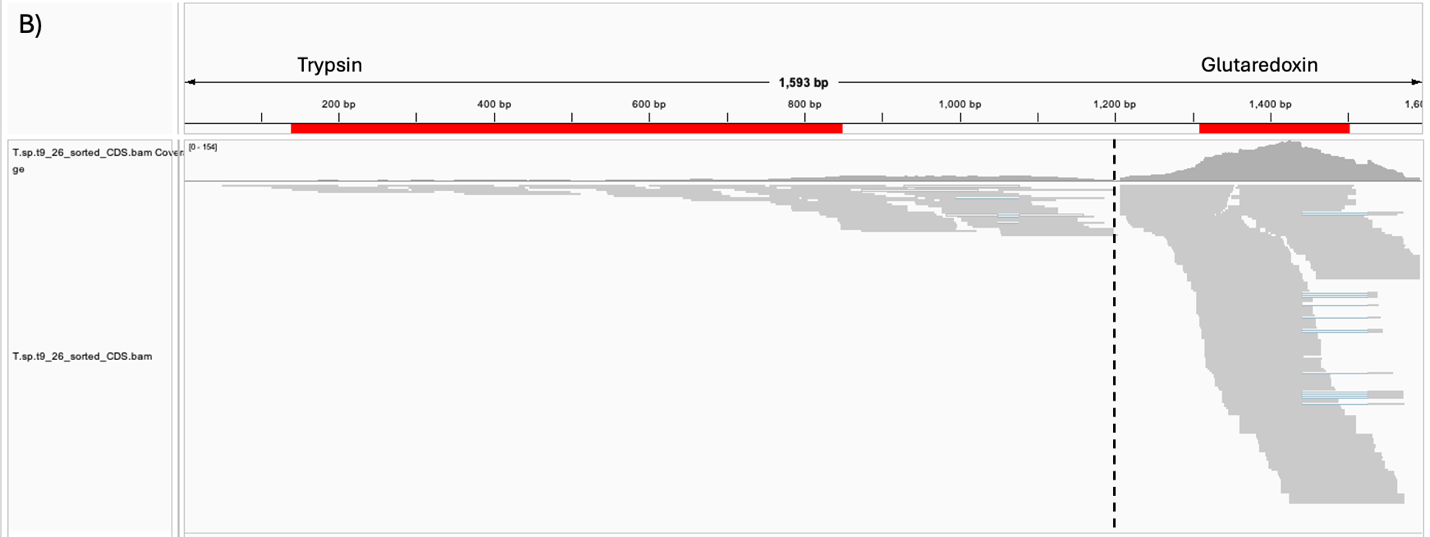
**


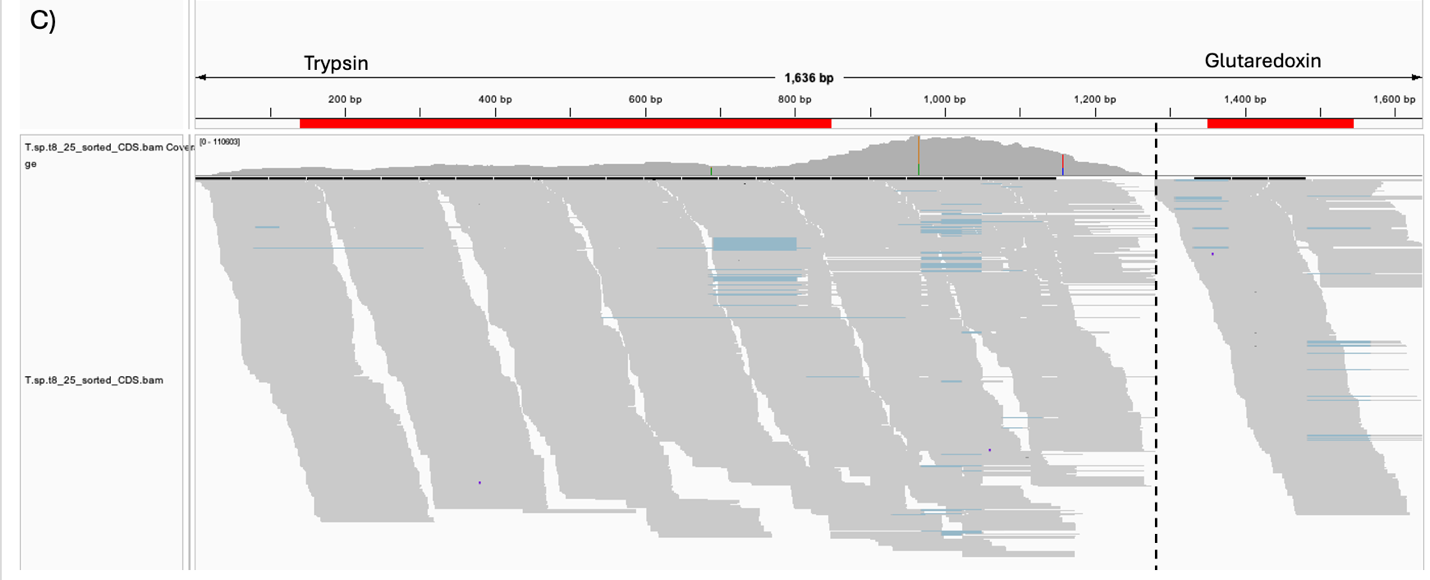


**
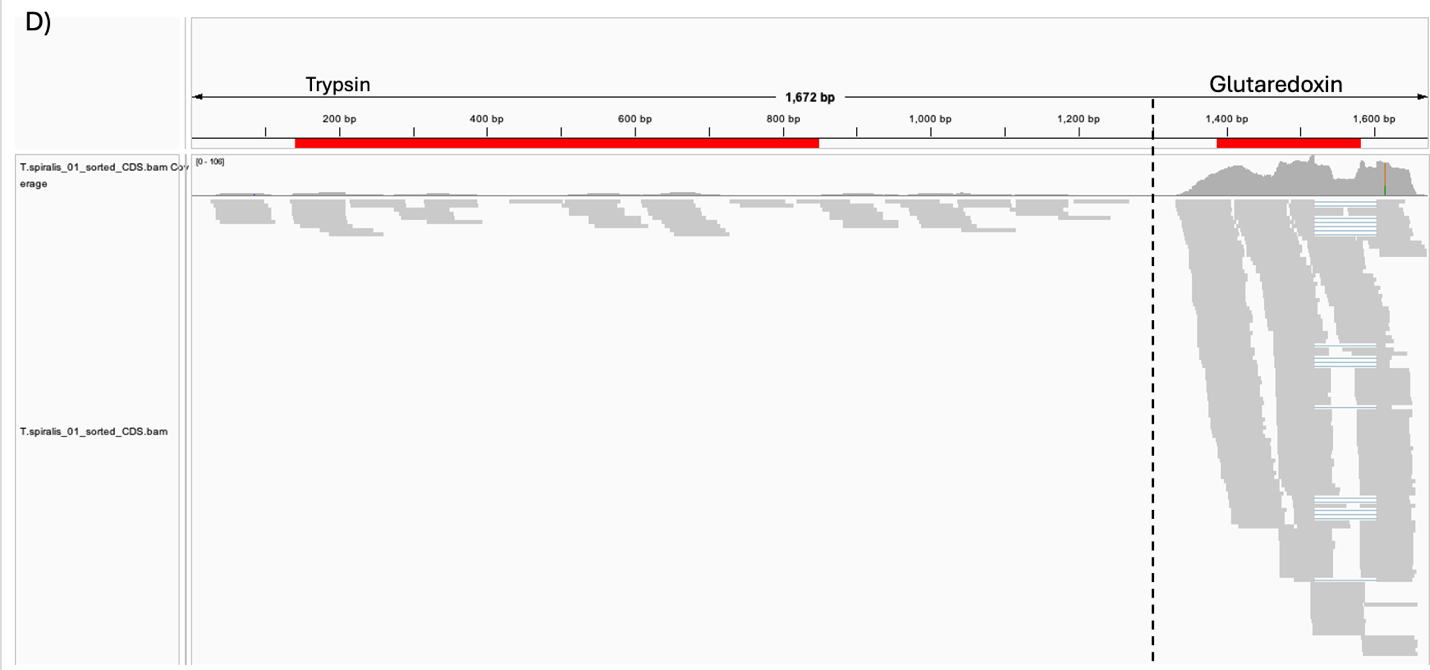
**

**
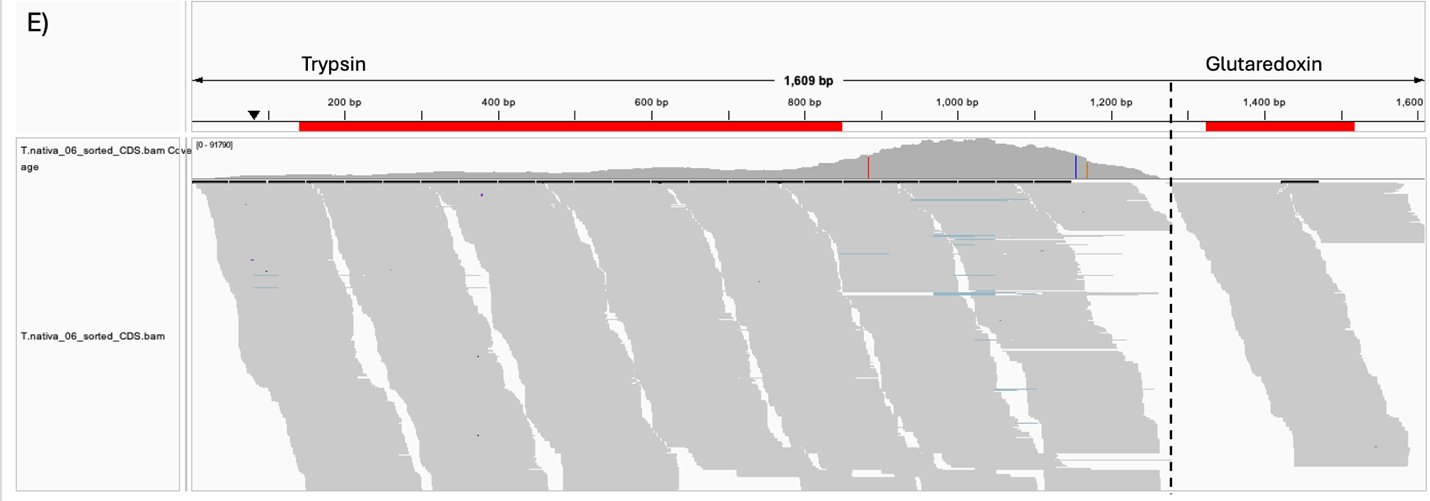
**


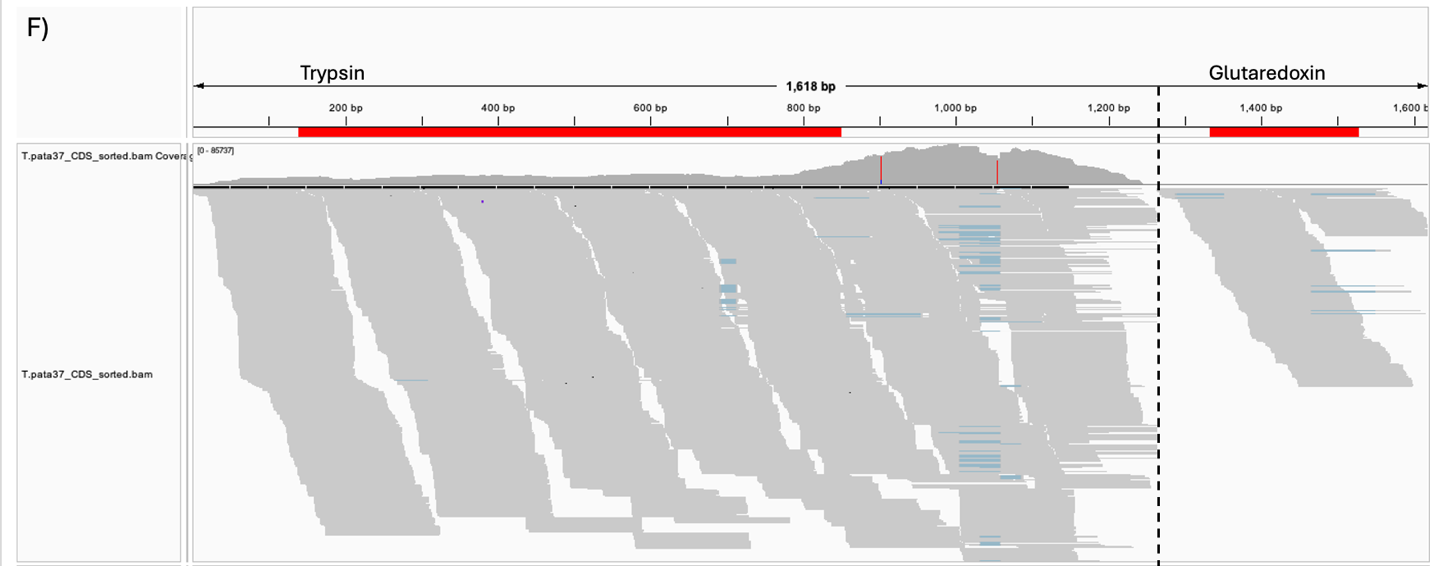


**
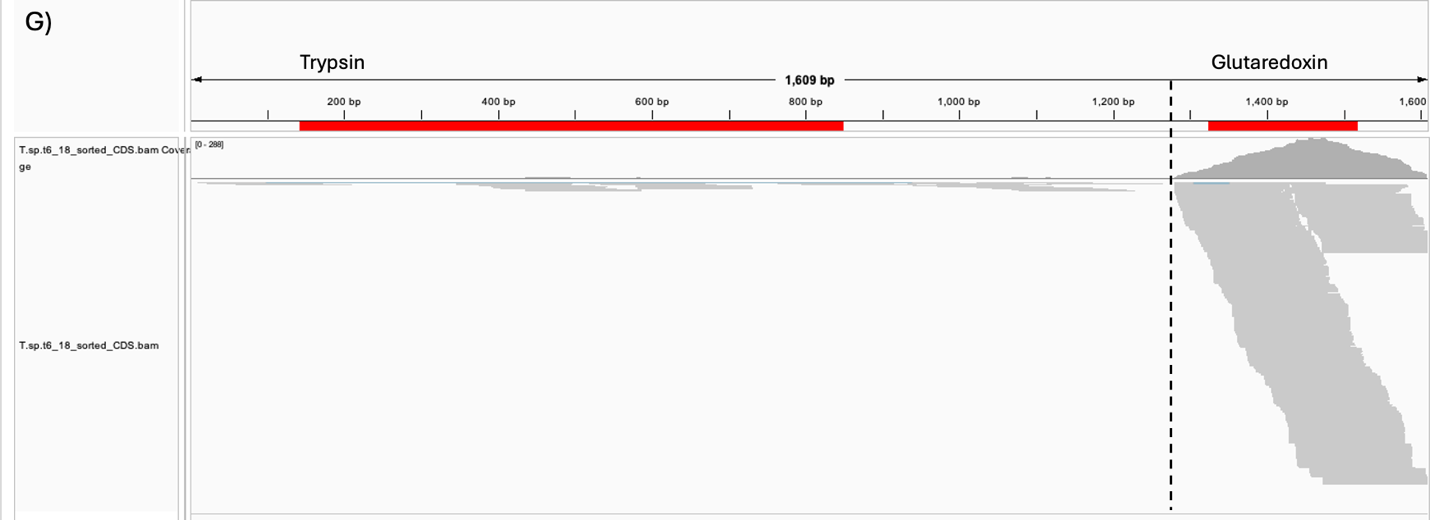
**

**
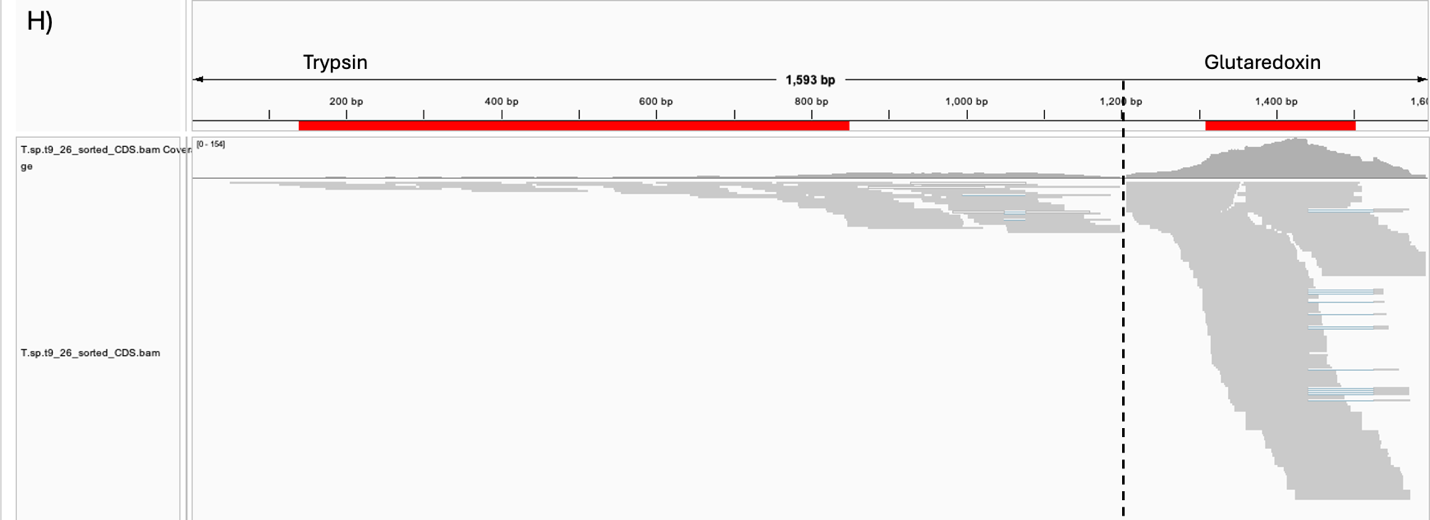
**

**
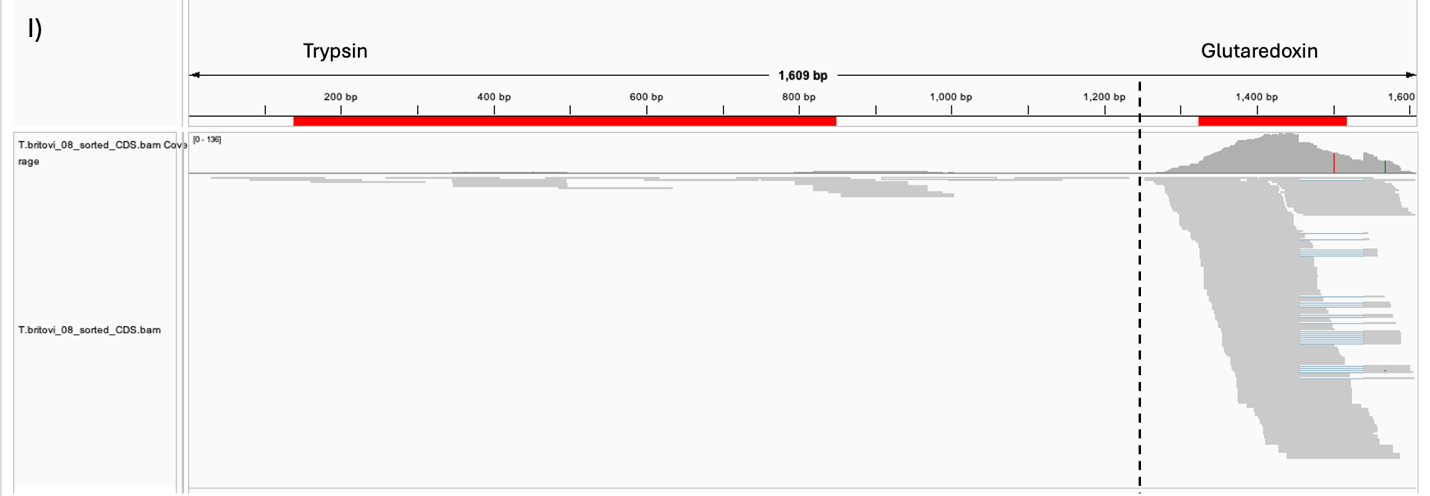
**

**
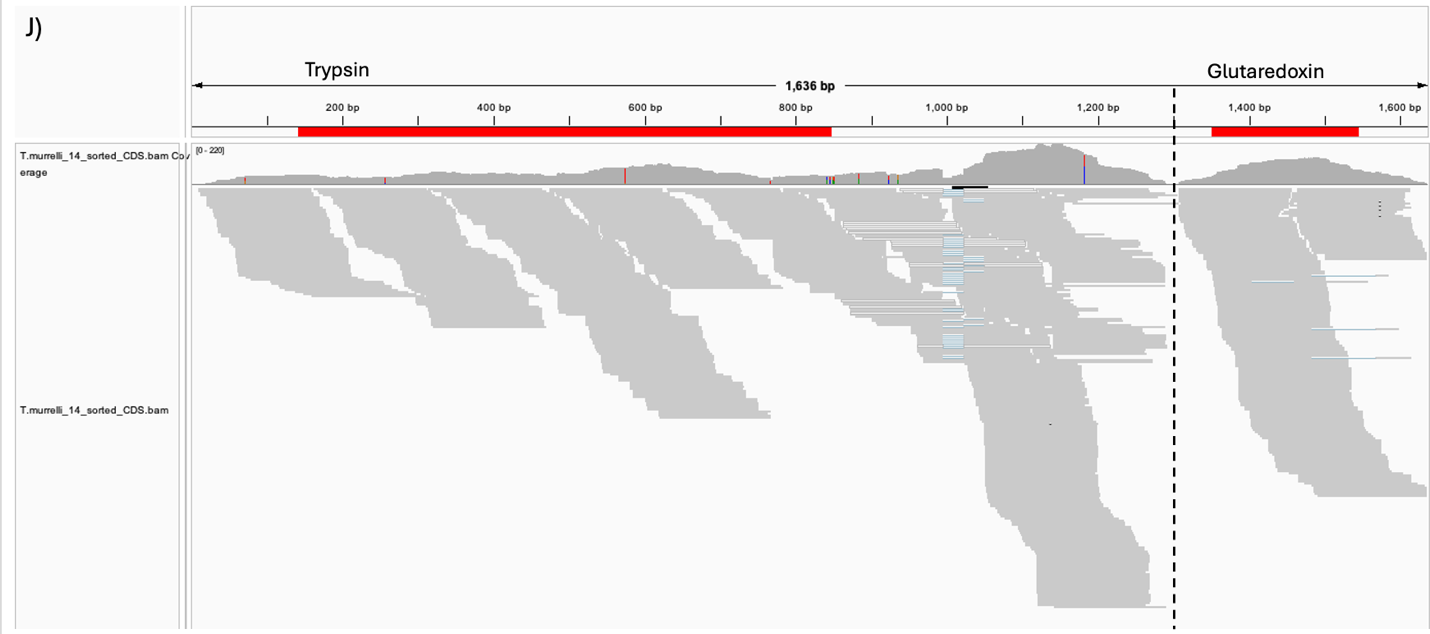
**

**
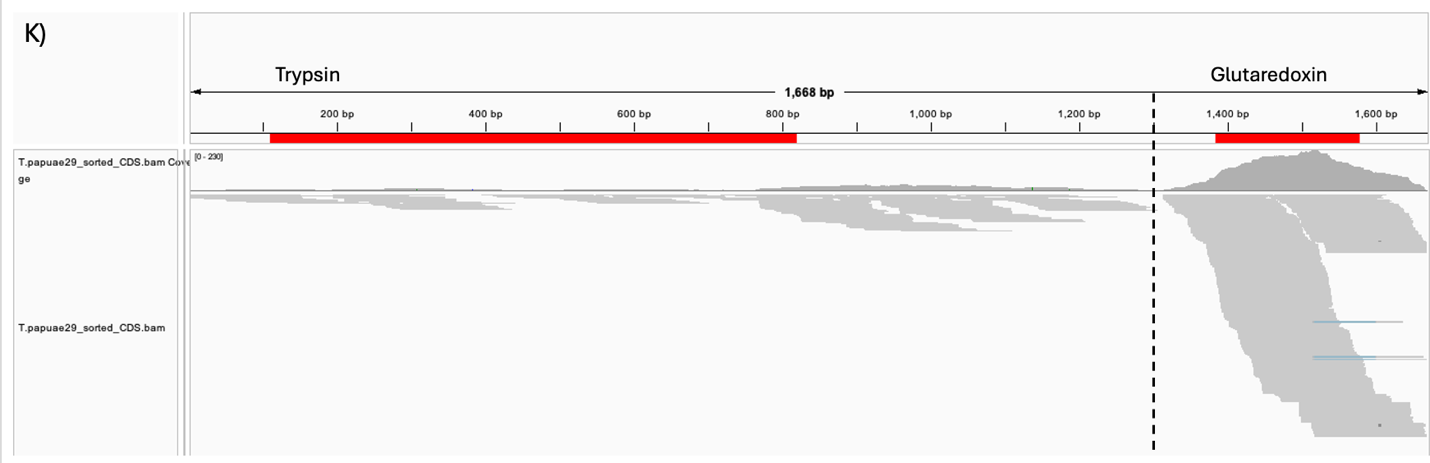
**


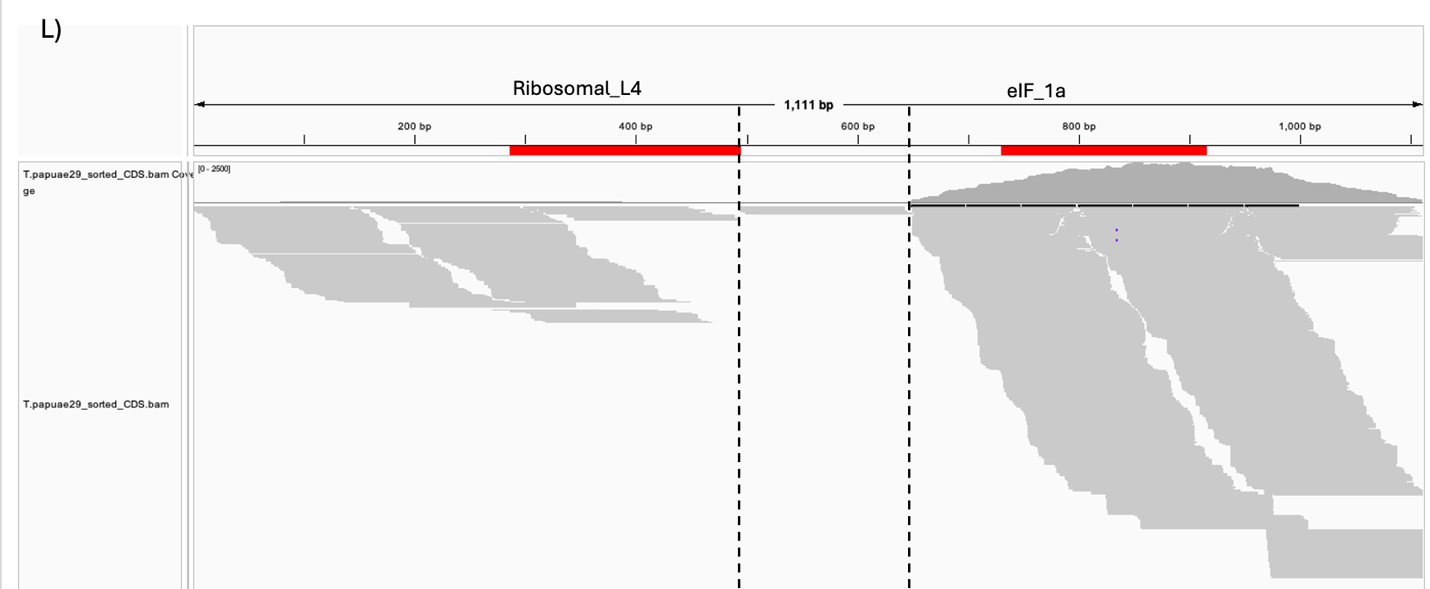


**
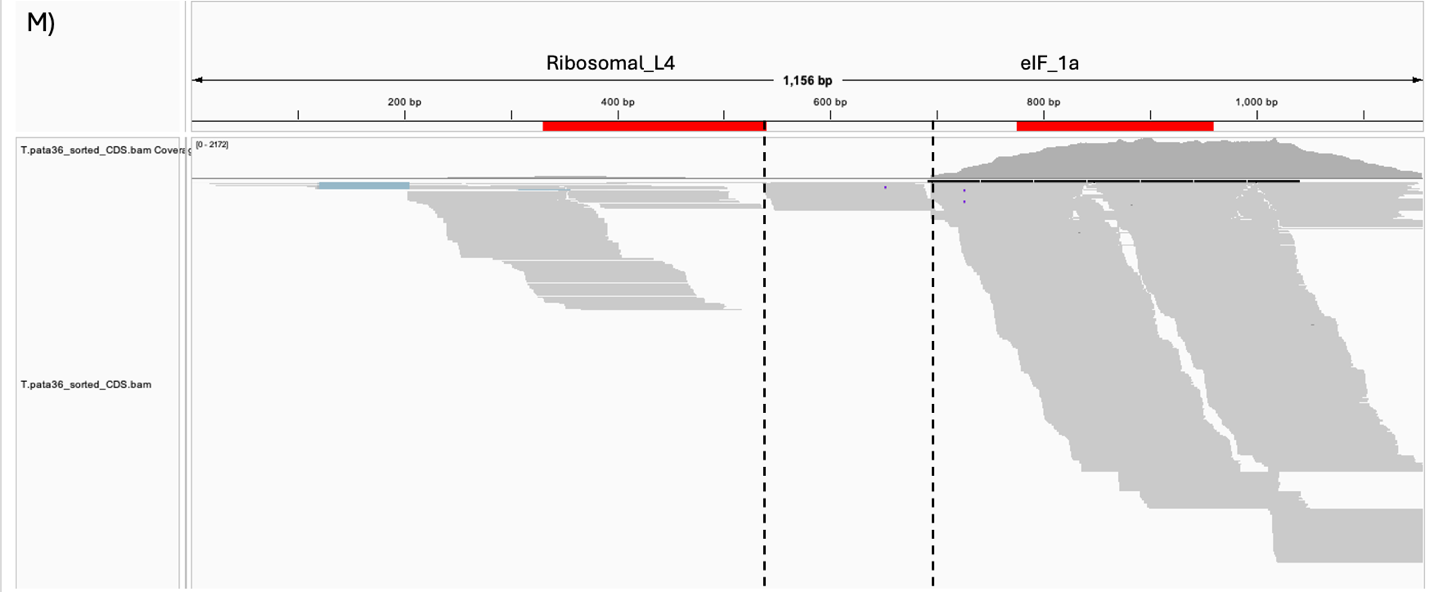
**

**
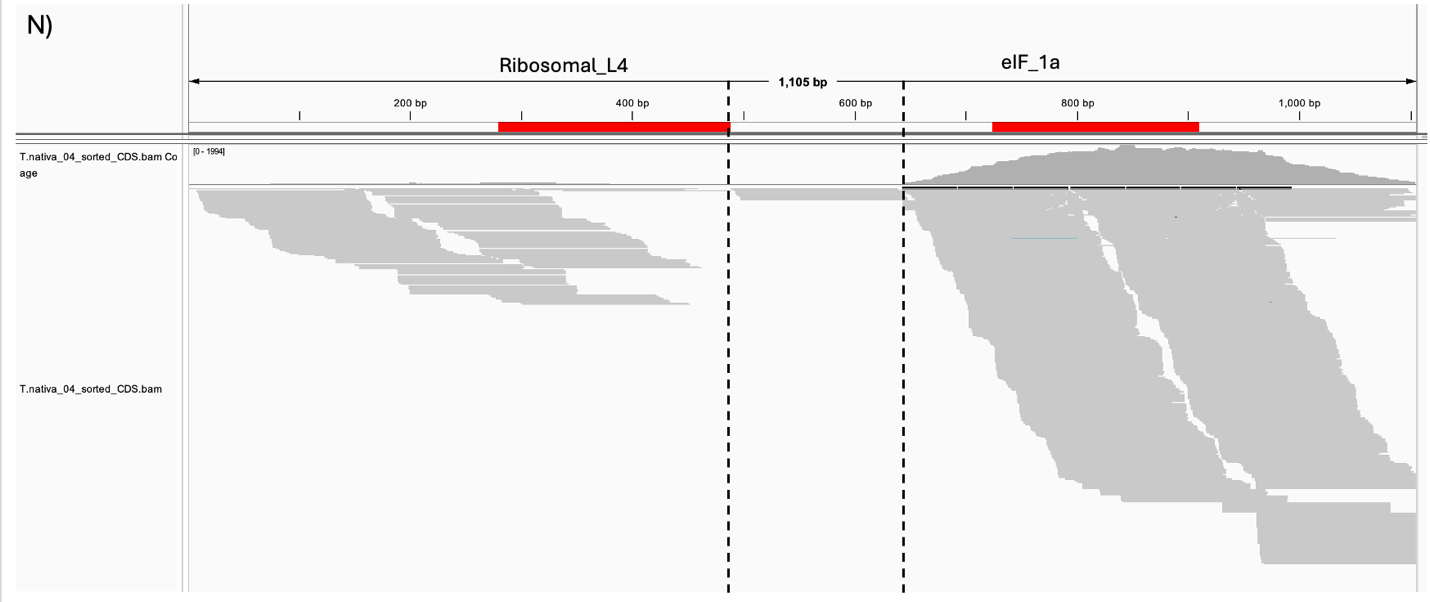
**

**
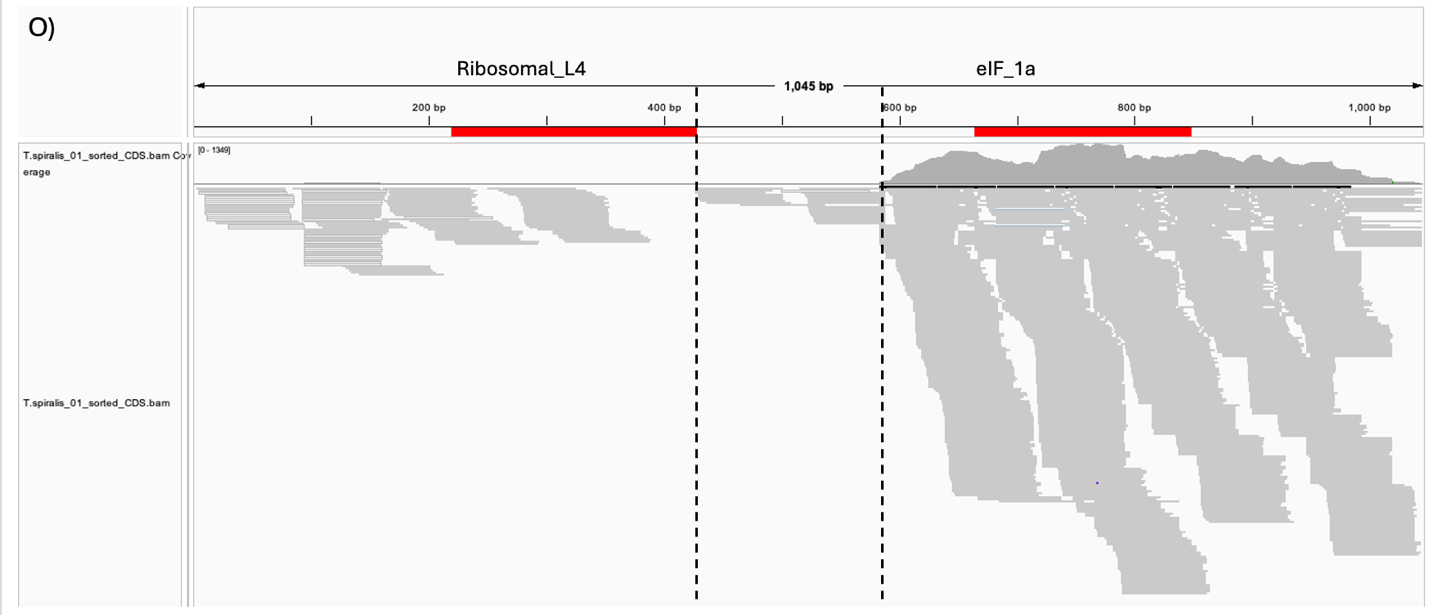
**

**
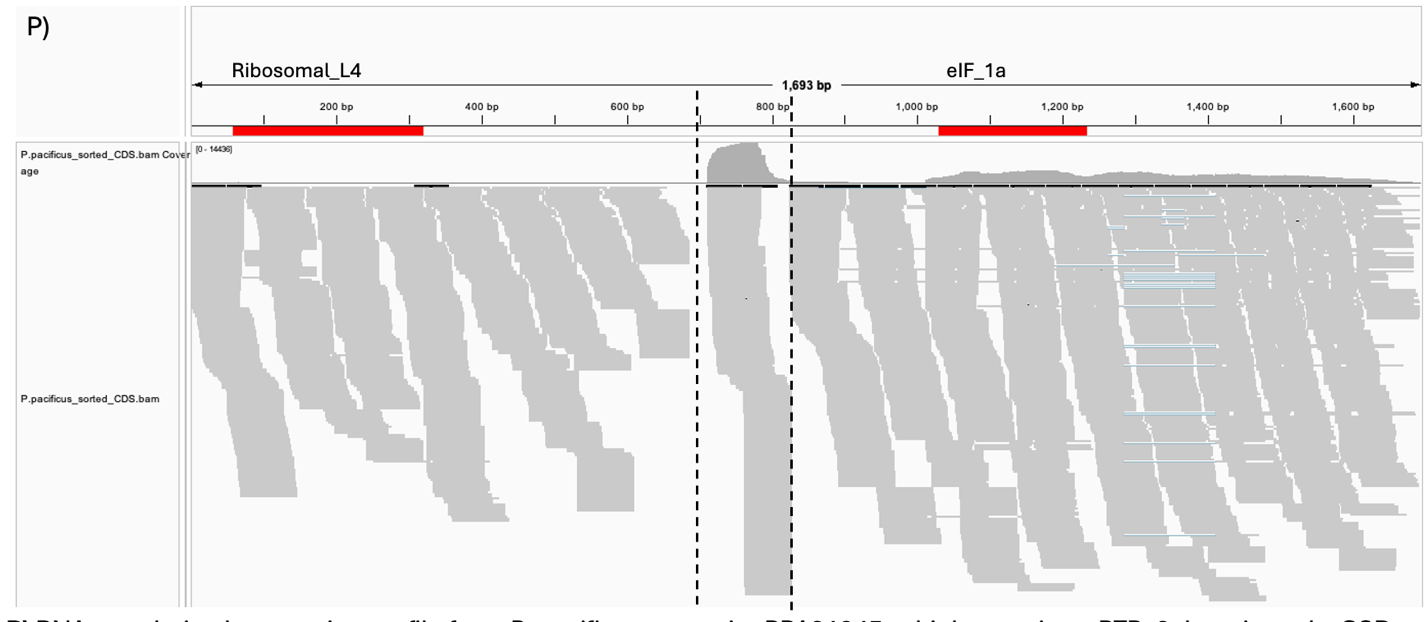
**

**
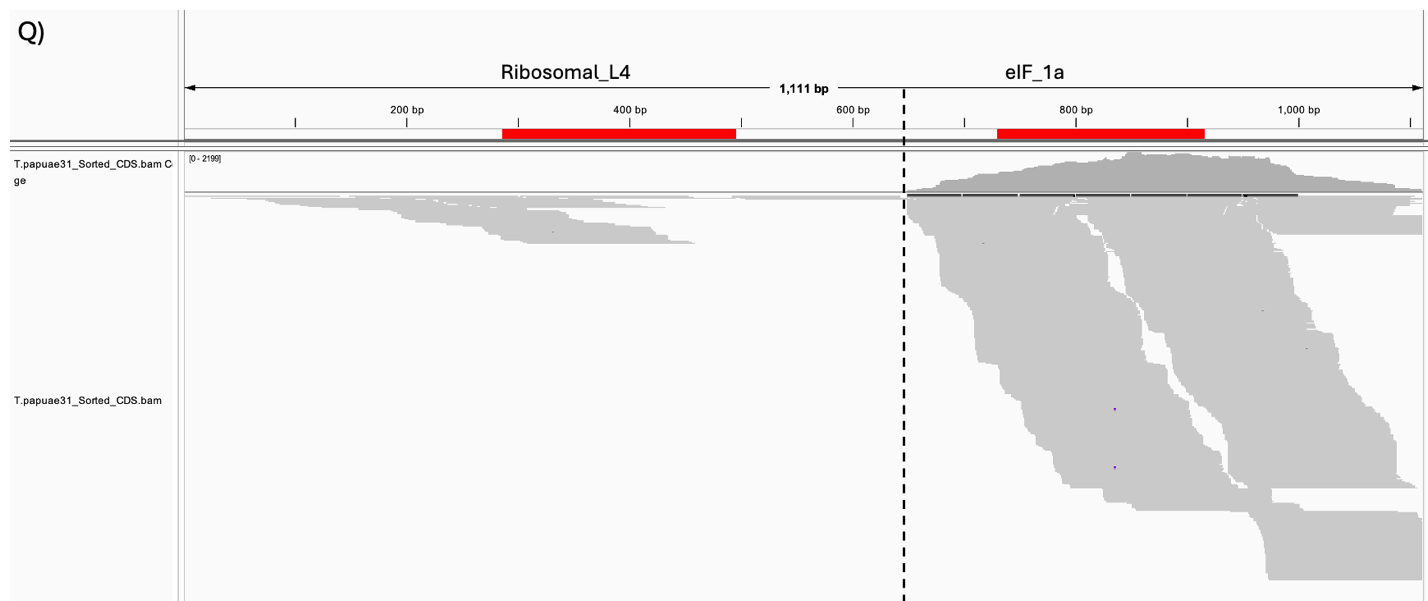
**

**
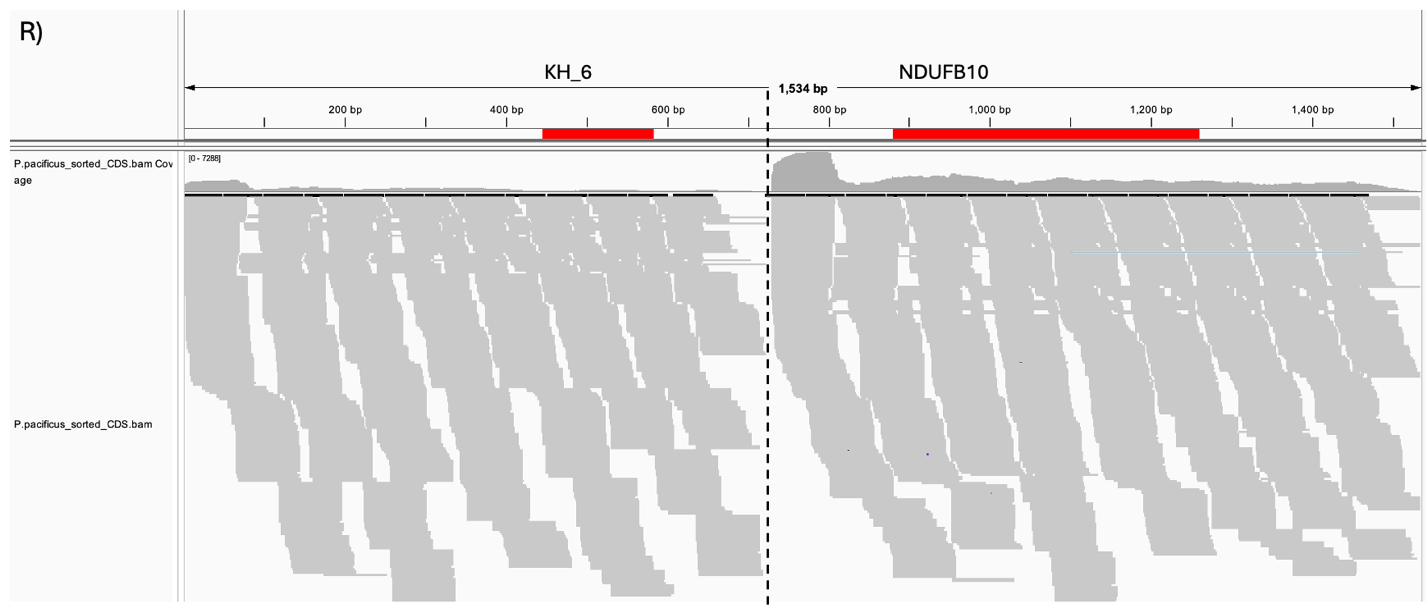
**

**
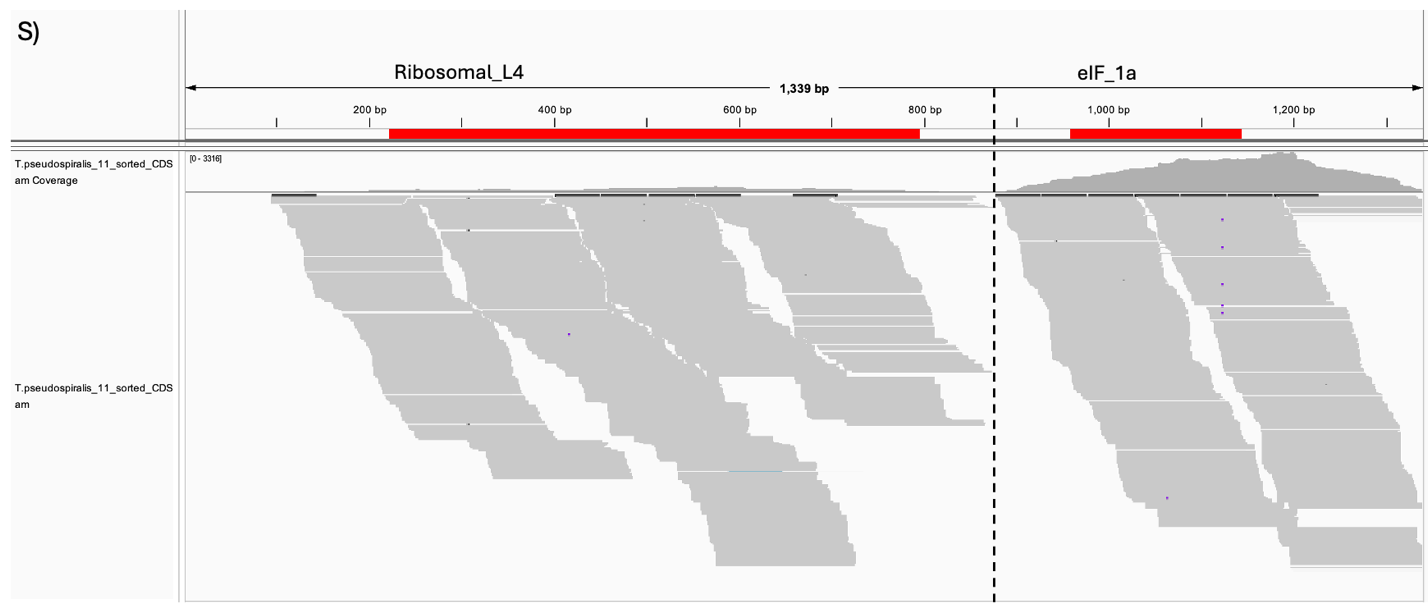
**

**
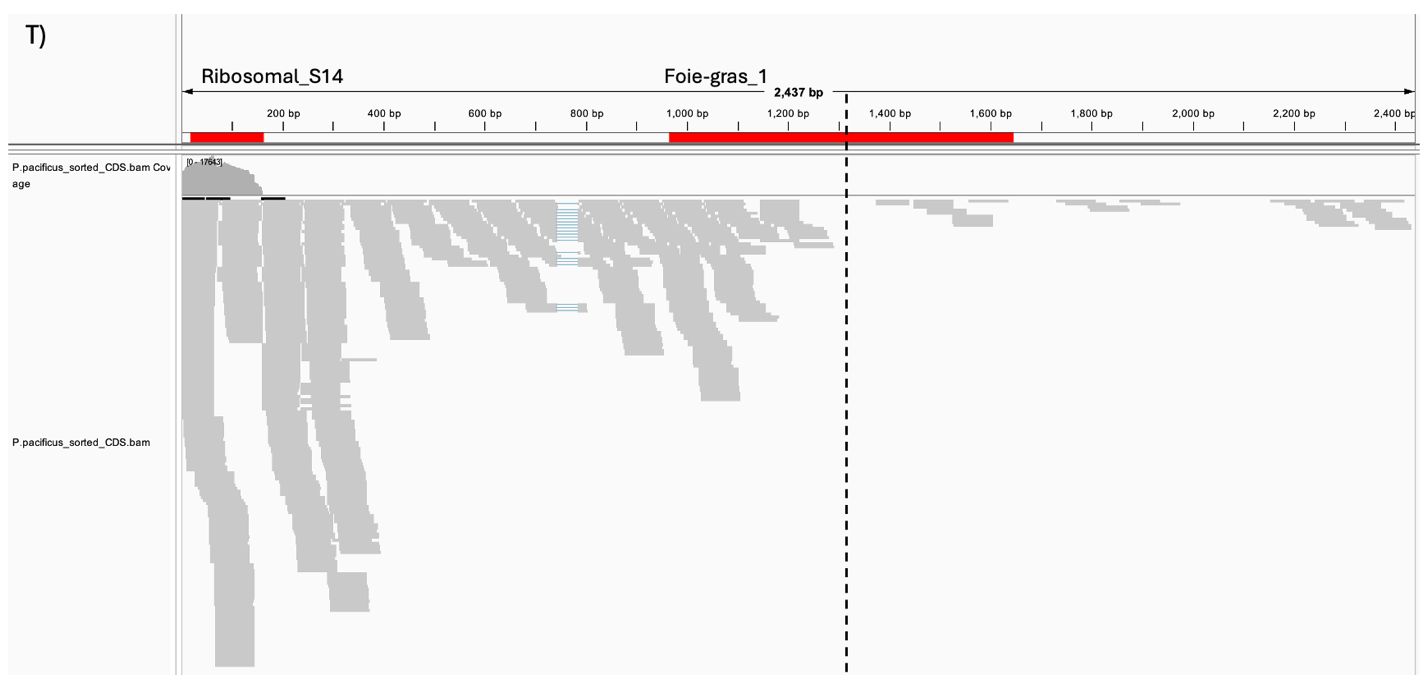
**

**
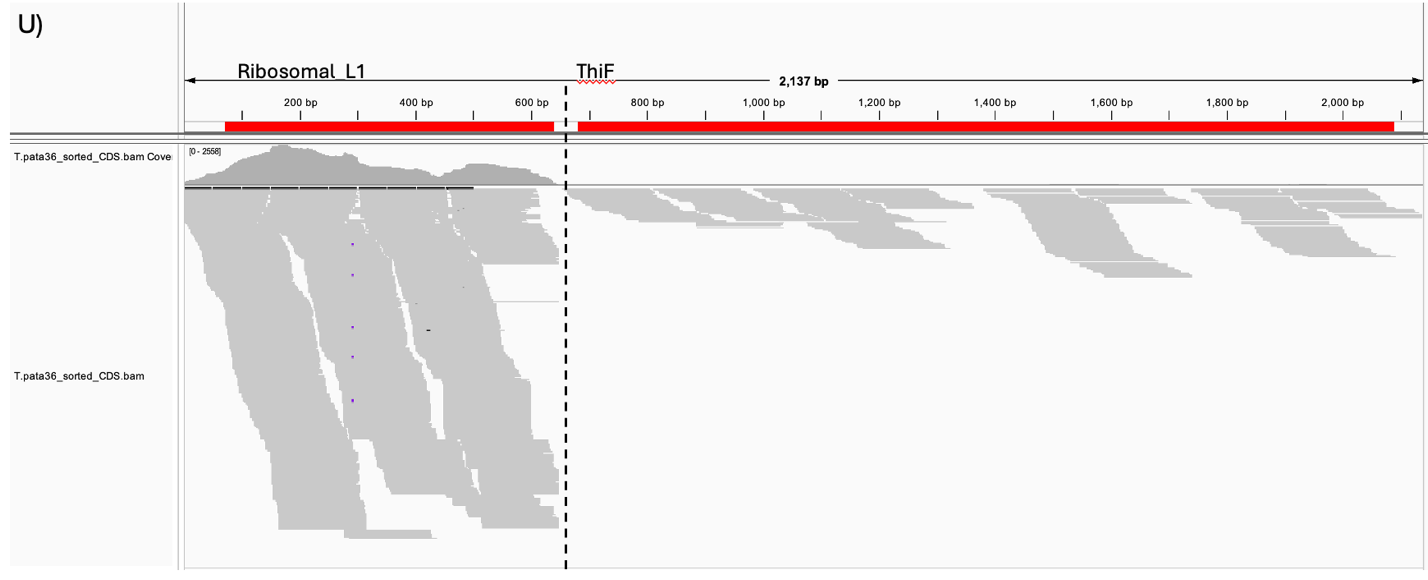
**

**Figure S9.** RNA-seq derived expression profiles for the 20 most distant points on the plot of average read counts of domain 1 and domain 2 for two-domain proteins unique to helminths. Domain locations are labelled red bars over the coverage track, and dashed lines indicate breakpoints in the expression profile where the number of reads drops to 0. A) Correlation between D1 and D2 expression levels in helminth-only two-domain proteins. Transcripts with the least correlated average read counts in domains 1 and 2 (circled) were selected for examination of coverage tracks using IGV. B) Adult *T. spt9* transcript T09_4248.1. This transcript contains a Trypsin domain and a Glutaredoxin domain, with an interdomain length of 458 bp. C) Newborn larva *T. spt8* transcript T08_3689.1, which contains a Trypsin domain and a Glutaredoxin domain separated by an interdomain region of 500 bp. D) Transcript T01_12234.1 in *T. spiralis* adult helminths. This transcript contains a Trypsin and a Glutaredoxin domain, with an interdomain region of 536 bp. E) Transcript T02_11024.1 from *T. nativa* newborn larva, containing a Trypsin and Glutaredoxin domain. The interdomain length of this transcript is 473 bp. F) Transcript T12_7676.1 in *T. patagoniensis* newborn larva. This transcript has two domains, Trypsin and Glutaredoxin, separated by an interdomain length of 482 bp. G) *T. spt6* transcript T06_7118.1 in mid-stage larva. This transcript contains two Pfam domains, Trypsin and Glutaredoxin. The interdomain length is 473 bp. H) Transcript T09_4248.1 from adult *T. spt9*. This transcript contains a Trypsin and Glutaredoxin domain separated by 458 bp. I) *T. britovi* transcript T03_13432.1 from mid-stage larva. Pfam identifies a Trypsin and Glutaredoxin domain in this transcript separated by 473 bp. J) Transcript T05_15466.1 from adult *T. murrelli.* It contains a Trypsin and Glutaredoxin domain, with an interdomain length of 500 bp. K) Adult *T. papuae* transcript T10_10371.1. Pfam identifies a Trypsin and Glutaredoxin domain in this transcript, with an interdomain length of 563 bp. L) *T. papuae* transcript T10_10800.1 in adult helminths. PfamScan identifies a Ribosomal_L4 domain, and an eIF_1a domain, separated by an interdomain length of 233 bp. M) T12_9092.1 from mid-larva stage *T. patagoniensis*. This transcript contains two Pfam domains, Ribosomal_L4 and eIF-1a, separated by a 233 bp long interdomain region. N) Adult *T. nativa* transcript T02_6187.1. This transcript contains a Ribosomal_L4 domain and an eIF-1a, with an interdomain length of 233 bp between them. O) Adult *T. spiralis* transcript T01_9087.1, which contains two Pfam domains, Ribosomal_L4 and eIF-1a. These are separated by an interdomain region of 233 bp. P) *P. pacificus* transcript PPA21345, which contains a BTB_2 domain and a CSD domain. These are separated by an interdomain region of 707 bp. Q) *T. papuae* newborn larval transcript T10_10800.1. This transcript contains a Ribosomal_L4 and eIF-1a Pfam domains, separated by an interdomain length of 233 bp. R) *P. pacificus* transcript PPA40165, which contains two Pfam domains, KH_6 and NDUFB10. The interdomain length is 296 bases. S) Adult *T. pseudospiralis* transcript T4A_1741.2, which contains two Pfam domains, Ribosomal_L4 and eIF-1a, separated by an interdomain region of 161 bp. T) Transcript PPA00675 from *P. pacificus.* This transcript has two Pfam domains, Ribosomal_S14 and Foie-gras_1, separated by an interdomain region of 800 bp. U) Transcript T12_1753.2 from the mid-larval life stage of *T. patagoniensis*. This contains Pfam domains Ribosomal_L1 and ThiF, with an interdomain length of 35 bases.
